# Understanding the Pre-Clinical Stages of Parkinson’s Disease: Where Are We in Clinical and Research Settings?

**DOI:** 10.3390/ijms26146881

**Published:** 2025-07-17

**Authors:** Camilla Dalla Verde, Sri Jayanti, Korri El Khobar, John A. Stanford, Claudio Tiribelli, Silvia Gazzin

**Affiliations:** 1 Liver Brain Unit “Rita Moretti”, Fondazione Italiana Fegato-Onlus, Bldg. Q, AREA Science Park, ss14, Km 163.5, Basovizza, 34149 Trieste, Italy; camilla.dallaverde@fegato.it (C.D.V.); ctliver@fegato.it (C.T.); 2 Department of Life Sciences, University of Trieste, 34139 Trieste, Italy; 3 Eijkman Research Centre for Molecular Biology, National Research and Innovation Agency of Indonesia (BRIN), Jakarta Pusat 10340, Indonesia; srij001@brin.go.id (S.J.); korr001@brin.go.id (K.E.K.); 4 Department of Cell Biology and Physiology, University of Kansas School of Medicine, Kansas City, KS 66160, USA; jstanford@kumc.edu

**Keywords:** models, pre-clinical, prevention, inflammation, redox-stress, vagus, α-synuclein, Braak, disease-modifying strategies

## Abstract

Parkinson’s disease (PD) is the second most common neurodegenerative disorder in the world. PD is characterized by motor and non-motor symptoms, but the diagnosis primarily relies on the clinical assessment of postural and movement abnormalities, supported by imaging and genetic testing. It is widely accepted that the disease process begins decades before the onset of overt symptoms. Emerging evidence suggests that neuroinflammation plays a central role in the pathogenesis of PD, particularly during the pre-clinical phase. Activated microglia, increased levels of pro-inflammatory cytokines, and persistent oxidative stress have all been associated with the gradual loss of dopaminergic neurons. Although earlier detection and diagnosis remain elusive, achieving these goals is crucial for advancing prevention and disease-modifying strategies. Clinical studies are ongoing. To fill the gap, research models that recapitulate the chronic disease progression of PD are crucial to test preventive and disease-modifying strategies. This review briefly summarizes clinical knowledge on PD as a starting point for improving research models. Furthermore, we will critically evaluate how the existing models have been utilized and highlight opportunities to overcome their limitations and enhance the translational relevance to clinical application.

## 1. Introduction

**Figure 1:**
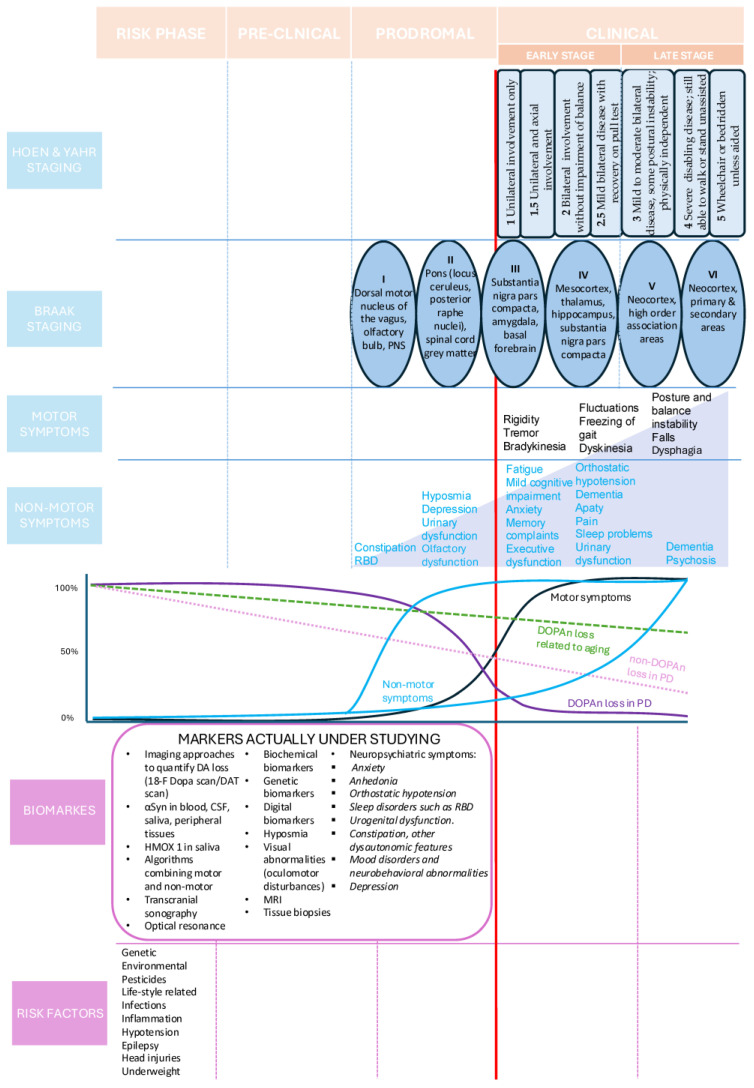
Graphical resume of clinical knowledge (light blue) and research features (pink) of PD progression. Abbreviations: PD: Parkinson’s disease; RBD: REM sleep behavior disorders; PNS: peripheral nervous system; DA: dopamine; DAT SCAN: dopamine transporter single-photon emission computed tomography; MRI: magnetic resonance imaging; αSyn: α-synuclein; CSF: cerebrospinal fluid; HMOX1: heme oxygenase 1; and DOPAn: dopaminergic neuron.

Despite the heterogeneity of motor and non-motor symptoms of Parkinson’s disease (PD), diagnosis is based primarily on the cardinal motor symptoms that include resting tremor, bradykinesia, rigidity, and postural instability. By the time a diagnosis is made, neurological damage, namely, dopamine neuron (DOPAn) loss in the substantia nigra (SN) and the consequent loss of dopamine (DA) in the putamen and caudate nucleus, have already advanced. The clinical symptoms observed at this stage are manifestations of pathological processes that began decades earlier (Figure 1) [1,2,3,4,5,6,7,8,9,10,11,12,13,14]. The progression of PD can be retrospectively categorized into the following four phases: (1) the clinical phase (post-diagnosis), (2) the prodromal phase, (3) the pre-clinical phase, and (4) the recently added risk phase (Figure 1) [1,12,15]. Among these phases, the clinical phase is the most extensively studied, both in human and research models. However, a significant limitation of focusing on the clinical stage is that observed molecular changes likely reflect the downstream consequences of the disease rather than the initial pathogenic mechanisms. As a result, such findings may not accurately capture the early biological processes involved in disease initiation. The other three phases encompass the pre-diagnostic stages of PD, which are critical for understanding its onset and for identifying potential preventive or disease modifying strategies. Due to the nonspecific nature of prodromal signs (see later) and lack of brain tissue from presymptomatic timepoints, human-based information on the molecular events underlying early disease progression in PD remains scarce. Thus, research models that recapitulate this time period are the key to solving this problem.

In this review we will first recap clinical knowledge and emergent hypotheses of PD onset and progression. We will then move on to how the PD models have complemented clinical research. We will conclude with suggestions for how basic science can fill knowledge gaps resulting from limitations of human studies.

## 2. What We Know About PD: The Clinical Readouts

A comprehensive review of PD is beyond the scope of this paper; thus, the following sections offer only a summary. Nevertheless, this background is necessary for modeling the disease and identifying potential readouts for research into the early stages of the disease.

### 2.1. Post-Diagnostic Phase

A PD diagnosis is based on the neurological exam focusing on hallmark motor symptoms [2,12,13,14], supported by imaging and family history, especially if genetic mutations are involved. Motor symptoms emerge with the loss of DA and DOPAn in the SN pars compacta and loss of DA in terminal regions in the caudate and putamen. The evolution of both motor symptoms and DA neuronal decline is described by the Hoehn & Yahr (H&Y) score (Figure 1) [16,17]. Autopsy data reveal rapid DA neuron loss in SN during the prodromal stage (see Section 2.1.1), with symptoms emerging with 50–70% of DOPAn loss in the SN, followed by a slower progression driven by multiple mechanisms [18,19,20,21,22,23].

Among the multiple pathologic mechanisms potentially responsible for DOPAn loss, protein aggregation (e.g., α-synuclein–αSyn, ubiquitin), proteasomal dysfunction, inflammation, mitochondrial dysfunction, reactive oxygen species (ROS) and nitrogen oxygen species (NOS), reduction in the anti-oxidant defense (GSH, reduced glutathione; SOD, superoxide dismutase), increased iron, altered calcium homeostasis, glutamate excitotoxicity, and reduced trophic factors, have all been described [2,23,24,25,26,27,28,29,30,31,32,33,34].

Based on the Braak hypotheses, linking αSyn accumulation and Lewy bodies (LBs)’ formation to disease progression, brain damage starts in non-DA structures of the lower brainstem or in the olfactory bulb (stages I–II), leading to the prodromal symptoms (see later); reaching the SN (stage III), resulting in the death of DOPAn and motor symptoms; and then spread caudally (stage IV–VI), contributing to more severe PD symptoms [19,35,36,37,38]. It has been suggested that αSyn may originate in the intestine and reach the brain via the vagus nerve (see risk phase). Based on this hypothesis, the clinical evaluation of αSyn in different peripheral sites (e.g., blood; cerebrospinal fluid, CSF; skin; and colon biopsies) is ongoing with the hope of achieving an early diagnosis of PD (see Figure 1) [12,39,40,41]. However, the pivotal role of αSyn has received increasing criticism [23,26,37,38,42,43,44]. Arguments against the synucleinopathy hypothesis include the following: (i) αSyn aggregates are not exclusive to PD [37,45]; (ii) Braak “pathology” (even staging 4 to 6) is reported in individuals without neurological impairment [46,47,48,49]; (iii) LBs are not always present in PD patients with motor symptoms [50]; (iv) the pattern of synucleinopathy described by Braak is inconsistent with observations, and there is no relationship between Braak’s stage and the clinical progression of PD described by the H&Y score [37,38,42,51]; (vi) Braak’s staging describes the accumulation of αSyn, not DOPAn loss, which has been reported to precede αSyn deposition in SN [23,52]; and finally, (vii) it is unclear whether αSyn-related pathological mechanisms correlate with neurologic dysfunction [2,26]. From a molecular point of view, αSyn is abundantly expressed in healthy brains, where it plays physiologic roles at the cell membrane and at the synapse [45,53], and it does not induce major histocompatibility complex II (MHC II) or persistent microglial activation [54,55]. αSyn misfolded fibrils can provoke an immunogenic response in the brain and aggregate inside the cells forming LBs [45,56]. PD has multiple manifestations, from rapidly progressing prominent non-motor signs that do not respond to treatments to mild progressing, predominant motor signs that do respond to drugs, and to mixed manifestations and prognoses [34,57]. Braak staging described progression in a specific subtype of PD with early onset and prolonged duration [38,43,58,59,60]. If synucleopathy is not the mechanism for DOPAn cell death and does not provide a reliable outcome measure, this has major translational implications for developing pre-clinical PD models.

The second most reported mechanism underlying neuronal loss is microgliosis and inflammation. Microgliosis, astrogliosis, and the presence of CD8^+^ and CD4^+^ adaptive immune T cells have been documented in the pons, basal ganglia, frontal and temporal cortical regions of PD human brains [33,61]. These may lead to the production of NOS, ROS, cyclooxygenase (COX) activation; release of the pro-inflammatory cytokine C-X-C motif chemokine ligand 12 (CXCL12); tumor necrosis factor alpha (TNFα); interferon-γ (IFNγ); and interleukin 6 (IL6) and interleukin 1β (IL1β) [62,63,64,65]. In line with this, increases in TNFα, IL1β, interleukin 2 (IL2), and interleukin 10 (IL10) are found in post mortem human PD brains and the serum of PD subjects [66,67,68]. These findings are likely more related to the late stages of the disease than to causative mechanisms [65]. However, glial cells also have homeostatic and protective roles during neurodegenerative conditions [69,70], clearing extracellular debris, and releasing trophic factors like the brain-derived neurotrophic factor (BDNF) and glial-derived neurotrophic factor (GDNF) [65]. Thus, glial activation is not synonymous with neuronal death per se. The dynamic role of glial cells during PD progression needs further study to distinguish cause from effect. Notably, serum TNFα levels in PD patients correlate with cognitive and mood decline [71,72], sleep disorders [71,72,73], and overall H&Y progression [24,72,73,74,75,76]. Moreover, infections may trigger PD symptoms, and PD-related genes overlap with inflammatory pathways, supported by reduced PD incidence or severity in patients undergoing anti-inflammatory treatment [22,61,69,70,77,78,79,80,81,82]. Thus, inflammation is hypothesized to work in two separate phases of PD, it likely precedes the clinical diagnostic phase, and may also drive neurodegeneration in the advanced stages of PD [83].

Mitochondrial dysfunction and redox imbalance are also key contributors to PD. Post mortem studies reveal mitochondrial complex-I activity and oxidative stress in post mortem PD brains [24,84,85,86,87]. A 4-fold higher expression of heme oxygenase 1 (HMOX1), a sensor for ongoing redox stress, has been detected in the SN but not in other brain regions of PD patients compared to age-matched controls [88,89,90]. HMOX1 hyperactivation may enhance ROS production by iron accumulation, a well-known contributor to neurodegenerative diseases [91,92,93,94].

Redox stress and inflammation are intimately linked [95,96], and neuro-inflammation and redox stress may lead to protein disruption and αSyn aggregation [62]. The time course of these processes is not fully understood.

**Figure 2:**
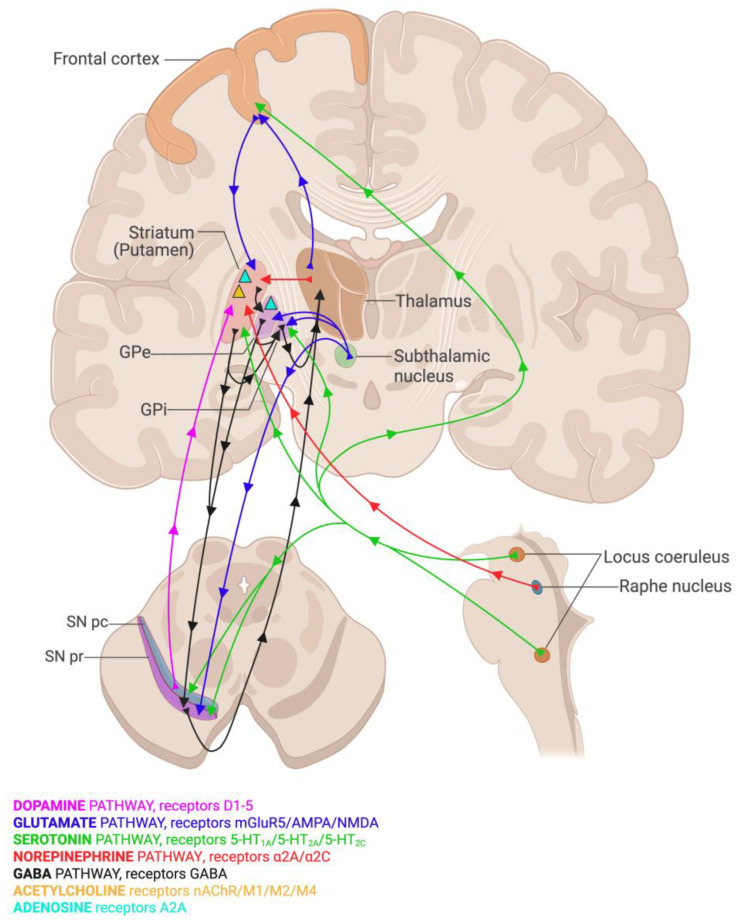
Interconnections between brain regions and neurotransmitters implicated in PD. Abbreviations: SNpc: substantia nigra pars compacta; SNpr: substantia nigra pars reticulata; GPi: internal globus pallidus; GPe: external globus pallidus; GABA: gamma-aminobutyric acid; mGluR5: metabotropic glutamate receptor 5; NMDA: N-methyl-D-aspartate receptor; AMPA: α-amino-3-hydroxy-5-methyl-4-isoxazolepropionic acid receptor; 5-HT_2A_: serotonin 2A receptor; 5-HT_1A_: serotonin 1A receptor; 5-HT_2C_: serotonin 2C receptor; D1–5: dopamine receptor 1-5; A2A: adenosine 2A receptor; nAChR: nicotinic acetylcholine receptors; M1: muscarinic acetylcholine receptor 1; M2: muscarinic acetylcholine receptor 2; M4: muscarinic acetylcholine receptor 4; α2A: α2A adrenergic receptor; and α2C: α2C adrenergic receptor. Created in BioRender. Dalla Verde, C. (2025) https://BioRender.com/ifgnt5e.

While SN lesions (DA and DOPAn loss) are responsible for symptoms at diagnosis (Figure 1), non-motor symptoms worsen and become predominant and potentially lethal in the late stage of clinical PD (Figure 1) [97,98,99,100]. Although decreased DA, DA transporters, and DOPAn in non-SN regions have been reported (Figure 2) [99,101], they do not fully explain non-motor symptoms (Figure 1). Instead, the degeneration of noradrenergic (NA), serotonergic (SE), and cholinergic (Chol) systems in other brain regions contributes significantly, with 30–90% loss in these neurons by the late stages [26,57,98,99,100,102,103,104,105,106,107,108,109,110]. These systems are linked to sleep disorders, non-DA-responsive gait and balance impairments, dementia, anosmia, and cognitive decline [98,101,108,109,111,112,113,114,115,116,117,118,119,120]. These circuits interact extensively with DA circuits [98,108,109,117,118,119,120].

Many non-motor symptoms have also been documented in the prodromal phase (Figure 1). Determining the mechanisms that contribute to the involvement of these neural circuits, from the prodromal phase to clinical PD, should help to reveal the real triggers of the disease.

#### 2.1.1. Prodromal Phase

Despite the age-related onset of clinical PD between 50 and 60 years of age, it is well known that the disease starts much earlier, possibly decades before the development of diagnostic symptoms [1,3,9,11,26,121]. At that stage, non-motor symptoms (Figure 1), such as olfactory impairment potentially strictly linked with cognitive problems [42], constipation, apathy, sleep disorders, anxiety, depression, REM sleep behavior disorders (RBD), hyposmia, fatigue, excessive daytime sleepiness, depression, and pain are usually already present, indicating that the neuropathologic mechanisms have begun [1,2,3,4,5,6,7,8,9,10,11].

Imaging studies have revealed early changes in neural circuits between patients with PD and healthy controls [10,122]. Decreased DA transporter binding has been measured in the nigrostriatal pathway in PD, with greater decrease in the terminal regions (caudate and putamen) than in the SN [123]. This suggests a retrograde degeneration of DA neurons in PD and opens a temporal window for improving the therapeutic efficacy. Bidirectional association between sleep disruption and dopaminergic loss is present. Aberrant αsyn deposition, neuroinflammation, and glymphatic system disruption are powered by sleep deprivation [124,125].

Recent experimental data suggest inflammation as a critical factor in PD (see later, in the risk phase section, and Figure 1). In agreement, physical activity throughout life shows neuroprotective action, possibly by decreasing systemic inflammation, or by maintaining the high level of BDNF, known to be decreased in PD [126,127]. Uric acid and deglycase J1 (DJ1) are under consideration as potential inflammatory-related biomarkers [10,128,129,130] (Figure 1). HMOX1 upregulation in plasma and saliva has been suggested as an early marker of PD onset [131,132,133]. Following Braak’s hypotheses, the evaluation of αSyn quantification in blood, CSF, and peripheral tissues is ongoing [10,39,122,128,134,135]. Pathological αSyn inclusions can also be found throughout the gastrointestinal tract up to 20 years before PD diagnosis, supporting Braak’s model of PD pathophysiology and making constipation one of the earliest identifiable prodromal features [124,125,136,137]. Abnormal αsyn deposition localized in the olfactory bulb of PD subjects has 95% of sensitivity and 91% of specificity compared to healthy elderly controls. In an MRI study on the anatomical changes in brain structures of an aged human population, PD patients showed a significant reduction in olfactory bulb volume compared to heathy controls. Microstructural degradation of the olfactory tract and substantia nigra are linked to the progressive dysfunction of the putaminal dopaminergic area. Moreover the olfactory function has been reported to correlate with the integrity of other neurotransmitter systems in PD [124,125] (Table 1).

Unfortunately, the predictivity of these markers is currently weak and additional research is needed [1]. Future efforts using open access datasets, bioinformatics, and “omics”-based approaches will likely play a role in optimizing markers to increase their predictivity [15,138,139,140,141,142,143,144,145,146,147]. The International Parkinson and Movement Disorder Society has established research criteria for identifying prodromal PD. These criteria include markers such as RBD, scores on the Unified PD Rating Scale (UPDRS), olfactory loss, constipation, excessive daytime sleepiness, symptomatic hypotension, severe erectile dysfunction, urinary dysfunction, and depression [148]. Given the absence of a clear neuroprotective or disease-modifying therapy for PD, the potential ethical issues of disclosing disease risk in a nonmedical context, and the uncertainty inherent in this emerging field, it is important to approach their use with caution (Table 1).

**Table 1:** Pre-clinical markers of the risk of developing PD under evaluation and suggestions from the models.

Marker Under Evaluation	Rational	Sampling	Supportive Molecular and/or Pathologic Evidences	References
αSyn presence	Higher in early PD patients. Suggested negative correlation with cognitive decline (CSF). GI: based on Braakt hypothesis.	Blood, CSF, GI	Still debated if its plasma level in PD subject is higher than in healthy individuals. CSF: usually reported lower in PD subject vs. healthy controls. GI: detected.	[10,39,122,128,134,135,136,137,148]
Uric acid	Higher levels are associated with a significantly decreased risk of PD.	Blood and urine	Anti-oxidant action.	[128]
DJ1	Higer in early PD patients.	CSF	Plays a role in mitochondrial dysfunction, oxidative stress, and chaperons activity.	[128]
HMOX1	Higher in early-on-set PD patients.	Plasma and saliva	Sensor of redox stress. Tentative protection by bilirubin production. By the other side: potentially dangerous by producing iron.	[68,131,132,133,149]
Bilirubin	Higher serum bilirubin level in early-stage PD.	Serum, plasma	Elevated serum or plasma bilirubin levels in PD may result from the overexpression of HMOX1, which leads to anti-oxidant and anti-inflammatory effects and may contribute to neuroprotection.	[150,151,152]
DA	Detecting DOPAn initial suffering that preceed DOPAn loss and the appearance of cardinal motor signs.	DAT-scan	Decrease in DA transporter binding.	[10,122,123]
Additional information from PD models				
DA	Retrograde degeneration of DA in the nigrostriatal system.	In models	Synaptic spine number and neurite length decrease after MPTP/rotenone treatment. VMAT2 blockage caused by toxins.	[153,154,155]
NE, SE, Chol reduced levels in the extra nigrostriatal areas	Hyposmia, REM sleep deficits and disturbances.	In models	Suggested dysfunction of the neurological circuits. Correlation among symptoms and neurotransmitter’s level.	[156,157,158,159,160,161,162,163]
Occurrence of gastrointestinal symptoms in prodromal and frank PD	Constipation, reduced intestinal motility.	In models	Alterations in the resident neuronal populations of the GI tract. Increased αSyn detected.	[164,165,166]

Legend: Clinical trials aim mainly in identifying and validating novel and precocious biomarkers for predicting the risk of developing PD. Models might add information on the correlation among prodromal molecular changes and prodromal symptoms, suggesting new markers to follow at clinical level with even minimally invasive approaches (imaging, GI biopsy). Abbreviations: αSyn: αSynuclein; PD: Parkinson’s Disease; CSF: cerebrospinal fluid; GI: Gastrointestinal; DJ1: deglycase J1; HMOX1: heme oxygenase 1; DA: dopamine; DOPAn: dopaminergic neurons; DAT-scan: dopamine transporter single-photon emission computed tomography; MPTP: 1-Methyl-4-phenyl-1,2178,3,6-tetrahydropyridine; VMAT2: vesicular monoamine transporter 2; NE: norepinephrine; SE: serotonin; Chol: cholinergic; and REM: rapid eye movement.

#### 2.1.2. Pre-Clinical Phase

Despite the initiation of pathophysiological processes, overt PD manifestations are not present in the pre-clinical phase. This gap suggests the possibility of identifying PD biomarkers and represents the primary target for modeling the disease in the laboratory (Figure 1). Due to the absence of any pathological sign, retrospective re-analyses of previous studies are the only approaches that might be applied to improve the specificity of biomarkers in clinics. Ethical issues accompany this strategy [167]; moreover, the absence of symptoms does not justify performing expensive analyses (e.g., 18-F DAT SPECT), which are seldom expensive. Even minimally invasive approaches (e.g., blood and saliva) are accompanied by ethical issues. Progress is being made by system biology tools in the identification of new genomic, transcriptomic, proteomic, lipidomic, and metabolomic molecules and new signaling pathways that may be quantified by minimally invasive approaches and that may be relevant to the pathogenesis [122]. Models may help by avoiding (in vitro models) or reducing (in vivo models) the ethical limitations, reducing the costs of the studies, but even more relevant giving access to central nervous system (CNS) biomolecular analysis in a dynamic form from the pre-symptomatic to the full-blown phases.

#### 2.1.3. Risk Phase

Determining the etiology of PD has been elusive, but many risk factors for PD have been reported. It is likely that PD (as the other age-related diseases) may develop as consequence of the lifelong accumulation of environmental and lifestyle “stressors” and their interactions with genetic factors [2,10,68,168] (Figure 1).

The list of the PD-related stressors is quite extensive, and it includes environmental factors (exposure to metals, pesticides and herbicides) [169,170,171]; the high consumption of milk and dairy products [172,173]; gut dysbiosis [5,79]; substance abuse; insulin resistance in metabolic syndrome; brain trauma; and the physiologic neuroinflammation accompanying aging [79,172,174,175,176]. Physical activity is also associated with a 34% decreased risk of PD, acting by increasing BDNF, and/or DA [79] or by decreasing the systemic inflammation [172,177], with systemic reduction in DA suggested preceding CNS DOPAn loss [122]. Notably, systemic DA cannot enter the brain but may be immunomodulatory [62].

PD incidence differs depending on ethnic origin, being higher in Hispanic people, then non-Hispanic White people, Asian people, and lower in Black people [178]. Genome-wide association studies identified 24 loci with clinically significant association with increased risk, and 11 variants showing a decrease in the risk of developing PD [179].

Some functional correlations between gene variants and environmental/lifestyle factors have been individuated [2]. Key molecular pathways presumed to be important in both familial and sporadic PD have been identified by fitting PD-related genes into common intracellular networks [180]. Recent perspectives on the etiology of PD suggest that the genes involved in PD may share biochemical mechanisms or signaling pathways with inflammatory pathways [69,70,74,77,78,81,121,181]. These include AMP/PKA/MAPK (cyclic adenosine monophosphate/ protein kinase A/ mitogen-activated protein kinases), NRF2 (nuclear factor erythroid 2–related factor 2), PPARs (peroxisome proliferator-activated receptors), NFκB (nuclear factor kappa-light-chain-enhancer of activated B cells), TLR4 (toll-like receptor 4), and inflammasome-NLRP3 (NOD-, LRR- and pyrin domain-containing protein 3) [69,174,175,182,183,184,185,186,187,188].

## 3. Where We Are in Understanding the Early Stages of PD Through Research Models

Research models for studying PD span different levels of complexity, from cell lines to non-human primates (NHPs). These models include the administration of toxins or proteins that result in various PD-like outcomes (see Table 2). Several reviews summarize the protocols used to mimic the disease, discussing the animal model [189]; route of administration [190,191,192]; dosages of the inducing agent [190,191,192]; the timing for developing motor signs and DA loss [193] and non-motor features [160,161,194]; and the effect of the animal age on the resulting phenotype [195].

In order to complement the existing literature, we will review the extent to which published studies address different stages of PD, with maximal interest in the accessibility to the pre-clinical stages, where research is available. The intention is to critically review whether the actual modeling of PD attempts to explore the sequence of events involved in the progression of the disease. This is a prerequisite to advance prevention and disease-modifying strategies.

**Table 2:** Common PD models: mechanisms and limitations.

Reserpine	Inhibition of VMAT2, which is expressed on synaptic vesicles of DOPAn, NE, and SE neurons and regulates the release of neurotransmitters [196]. Accordingly, dysfunction of these circuits in PD has been reported [161,189,197]. This model reproduces motor signs [161,198,199,200]; DA loss [189,199,200]; non-motor signs [161]; αSyn presence [199]; mitochondrial dysfunction and redox stress [155,161,199,201]; autophagy [155,161,199,202]; and inflammation [161,199].
6-OHDA	Production of ROS and inhibition of mitochondrial respiratory chain complexes I and IV [203]. Reduces GSH and SOD reduction. Increases glutamate (Glut); astrogliosis, autophagy, and proteasomal dysfunction induction [161,204]. Does not cross the blood–brain barrier (BBB), so intracranial injections are necessary. High mortality rate when administered bilaterally. Endogenous production of 6-OHDA has been reported in the brains of PD patients [55,189,203,205]. This model reproduces motor signs [55,156,161,189,198,199,205,206,207,208,209,210,211,212,213,214,215,216,217,218,219]; DA loss [55,156,189,198,199,205,206,207,208,209,210,211,212,213,214,215,216,217,218,219,220,221,222]; non-motor signs [156,160,161,207,211,212,213,218,223,224]; non-DA loss [156,160,189,212,213,214,216]; αSyn presence [208] and non-detected [55,189,199,205,206]; mitochondrial dysfunction and redox stress [161,199,209,210,221,223,225,226,227,228,229,230,231,232,233]; autophagy [161,199,234]; and inflammation [161,199,207,217,219,220,235].
MPTP	MPTP crosses the BBB due to its lipophilic nature. In the brain MPTP is converted to MPTP^+^ by the glial MAO-B, spontaneously oxidized to MPP^+^, then taken up into DA neurons by DAT [198,236,237]. MPTP may also be administered, but the positive charge reduces its brain bioavailability. Stereotaxic injection provide better results [193]. In cells MPP^+^ acts by accumulating in VMAT2 vesicles, leading to DA release and toxic auto-oxidation. MPP^+^ also binds to mitochondrial NADH, decreasing ATP production and inhibiting complex I with ROS production [208]. MPTP increases Glut, induces astrogliosis, microgliosis and cytokine release [238,239]. Rats are resistant to MPTP, thus requiring higher doses, and presenting high mortality rates secondary to massive loss of neurons [193,208,240]. Acute doses in MPTP models like mice, rats, zebrafish, and monkeys lead to a quick decrease in ATP, potentially resulting in rapid DOPAn death by apoptosis and necrosis [189,205,208,237], possibly being too rapid to allow proper access to each stage. Chronic administration of low doses in PD features in a small percentage of animals [189,193,197]. This model reproduces motor signs [156,161,189,199,205,208,214,237,241,242,243,244,245,246,247,248,249,250,251,252,253,254,255,256]; DA loss [55,156,189,198,199,205,206,208,214,238,241,242,243,244,245,246,248,249,250,251,252,254,255,256,257,258]; non-motor signs [156,160,161,241,242,250,255,256,259,260,261,262] and non-detected [249]; non-DA loss: [156,160,189,214,242,247,248,252,255,261,262] and non-detected [249,250]; αSyn presence [55,189,198,199,206,208,241,242,261,262] and non-detected [205,249]; absence of LBs [205,249,261,262]; mitochondrial dysfunction/redox stress [161,189,199,242,244,245,257,263,264,265,266,267,268,269,270]; autophagy [161,199,234]; inflammation [161,199,238,241,242,243,257,258,261,262,267,269]; and GI [164].
Paraquat	Agricultural herbicide linked to PD in farmers; not DA-specific. Paraquat induces mitochondrial dysfunction and ROS production [84], reduces GSH and thioredoxin, leads to lipid, protein, and DNA damage, inflammation, autophagy, and proteasomal dysfunction [189,197,205,237]. Potentially toxic to the liver, kidney, and lungs; high mortality rate [208]. This model reproduces motor signs [55,156,161,198,199,205,206,208,271,272,273,274]; DA loss [55,156,198,199,205,206,208,272,273,274]; non-motor signs [161,272]; non-DA loss [156,271,274]; αSyn presence [55,199,206] and non-detection [205,208]; but with the absence of LBs [55,189]; mitochondrial dysfunction and redox stress [161,189,199,263,271,274,275,276,277]; autophagy [161,199]; and inflammation [161,199].
Rotenone	Agricultural herbicide and insecticide linked to PD. Acts similarly to paraquat on mitochondria, redox state [79,84,161,189,193,205,237,278,279], and inhibiting proteasome activity [208], but it does not activate the caspase pathway [280]. Recently, low doses have been shown to have a pro-inflammatory effect [281,282,283]. It is water and alcohol insoluble, rapidly degraded by light, and metabolized in the liver and gastric mucosa. Thus, its usage in vivo is somehow difficult [192]. Acute doses may lead to systemic toxicity and necrosis in the brain [284], with potential lethality [192]. Heterogeneity in the time scale of symptom development and low percentage of animals developing the disease are scientific and ethic problems, overstepped recently by the use of older animals and adjuvants [192,195,285]. Rotenone reproduces motor signs [55,156,161,189,198,199,205,206,208,282,283,285,286,287,288,289]; DA loss: [55,156,189,198,199,205,206,208,282,283,285,286,287,288,289]; non-motor signs [156,161,208]; non-DA loss [156,206]; αSyn presence [55,189,199,206,282,285,288] and not detected [205,208]; LBs [189,288]; mitochondrial dysfunction and redox stress [161,199,263,266,286,289,290,291]; autophagy [161,199,292,293,294,295,296]; inflammation [154,161,199,282,283,288,289,290,294,297]; and gastro intestinal manifestations (GI) [156,165,166].
LPS	Pro-inflammatory [193], but also induces mitochondrial dysfunction and redox stress [161,194,199]. Largely used in recent years to explore the most recent hypotheses that inflammation is important in PD outcome. LPS reproduces motor signs [67,161,194,199,298,299,300,301,302,303,304,305,306,307]; DA loss [194,199,298,299,302,303,304,305,306,307,308,309]; non-motor signs [161,194,300,301]; non-DA loss [194,298,299,300,301,302,305]; αSyn presence [194,199,302,305,309] and non-detected [194]; mitochondrial dysfunction and redox stress [161,199,298,300,301,305,306,308]; autophagy [161,199]; inflammation: [67,161,199,298,299,302,303,304,305,306,307,308]; and GI [307]
αSyn	Protein physiologically present. It can be oxidated, modifying its folding. Misfolding provokes the formation of amyloid fibrils that will result in forming the LBs [45,53,54,55,56], motor signs [310,311,312]; DA loss [54,310,311,312]; αSyn presence [54,310,311,312]; LBs [310]; inflammation [54,310,312].
Genetic models	Multiple models based on multiple genetic variants for each PD gene exist. Large variability of the PD-like features is reached in all genetic animal models. The variability is due both to the genetic variant reproduced and the promoter that controls the gene of interest expression [199,313,314,315]. PARK1/4(SNCA) * has been reported to reproduce motor signs [55,161,189,198,199,205,206,313,314]; DA loss [55,160,198,314] and non-detected [189,199]; non-motor signs [160,161,314]; non-DA loss [160,313]; αSyn presence [55,199,206,313,314] and non-detected [206,313,314]; mitochondrial dysfunction [161,199]; redox stress [161]; autophagy [161,199]; GI [314,316]; inflammation [161,199]. PARK8 (LRRK2) ** has been reported to reproduce motor signs [55,189,198,206,313] and non-detected [161,199,205,314]; DA loss [198,314] and non-detected [199,205]; non-motor signs [161]; non-DA loss [313]; αSyn presence [198] and non-detected [55,189,205,313]; absence of LBs [206,313]; mitochondrial dysfunction [161,199]; redox stress [161]; autophagy [161,199]; GI [314,317]; and inflammation [161,199]. PARK2 (PRKN) *** has been reported to reproduce motor signs [199,313,314]; absence of DA loss [314]; non-motor signs; non-DA loss [189,199,206,314]; αSyn presence [314] and non-detected [189,206,313]; redox stress [199]; GI [199]; and inflammation [189]. PARK7 (DJ1) has been reported to reproduce motor signs [313,314] and non-detected [199,205,206]; DA loss [314] and non-detected [189,199,205,206]; non-DA loss [313]; αSyn presence [314] and non-detected [199,205,206]; absence of LBs [313]; mitochondrial dysfunction [199]; inflammation [199]. PARK5 (UCHL1) has been reported to reproduce motor signs [198,199,206]; DA loss [198]; non-motor signs [314]; absence of αSyn [199]; absence of LB [206]. PARK6 (PINK1) has been reported to reproduce motor signs [313,314] and non-detected [205]; DA loss [189,314] and non-detected [205]; non-motor signs [314]; non-DA loss (only in mice) [313]; αSyn presence [314] and non-detected [189,205,313]; absence of LBs [313]. VMAT2 has been reported to reproduce motor signs [160]; DA loss [160]; non-motor signs [160]; non-DA loss [160].

This table recaps the major outcomes and weaknesses in modeling PD based on the disease inducer. Abbreviations: PD: Parkinson’s Disease; VMAT2: vesicular monoamine transporter type 2; DA: dopamine; NE: norepinephrine; SE: serotonin; 6-OHDA: 6-Hydroxydopamine; ROS: reactive oxygen species; GSH: reduced glutathione; SOD: super oxide dismutase; Glut: glutamate; MAO-B: glial monoamine oxidase B; MPP^+^: 1-methyl-4-phenylpyridinium; MPTP: 1-Methyl-4-phenyl-1,2,3,6-tetrahydropyridine; DAT: dopamine transporter; NADH: nicotinamide adenine dinucleotide-coenzyme 1; ATP: Adenosine triphosphate; DOPAn: dopaminergic neurons; BBB: blood–brain barrier; SNCA: synuclein alpha gene; PRKN: parkin RBR E3 ubiquitin protein ligase; DJ1: deglycase J1; UCHL1: ubiquitin carboxyl-terminal hydrolase isozyme L1; PINK1: PTEN-induced kinase 1; LRRK2: leucine-rich repeat kinase 2; αSyn: α-synuclein; LB: Lewy body; GI: gastrointestinal signs; LPS: lipopolysaccharides. * Depending on the promoter. Synucleinopathy, neuronal degeneration and motor and/or non-motor signs are present only with mPDGFb/Pitx3/mPrp (hA53T)/mThy1 (hwt) promoters. Synucleinopathy and motor/non-motor signs (but not neurodegeneration) are present under PAC (hA53T) control. Synucleinopathy and motor signs (but not neurodegeneration and non-motor signs) in mPrp (E46k)/rTH (wt1–120)/mTHy1 (hsyn-A30P). ** Synucleinopathy and non-motor signs under Cam-TA /PAC (hA30P) in rodents. Synucleinopathy, neuronal degeneration and motor signs in mTh1 (A53T) in rodents [314]. *** Only the bacterial artificial chromosome (BAC) model shows synucleinopathy, neuronal degeneration and motor signs in rodents [314]. NB: In zebrafish neurodegeneration and motor deficits occur in the absence of synucleinopathy for SNCA, LRKK2, DJ1, PARKIN and PINK mutational variants [314].

### 3.1. Modeling PD

Based on clinical and pathological features, a successful animal model of PD should include DA/DOPAn loss, motor deficits, αSyn, and/or LBs formation. Protein misfolding, inflammation, and redox impairment are the major pathologic mechanisms suspected in clinic and frequently investigated in PD models (in vitro to in vivo) (Table 2, Table 3 and Table 4).

#### 3.1.1. Modeling the Early Stages of Clinical PD

Motor symptoms (rigidity and akinesia) together with a DOPAn loss up to 75% in SN, and they may be presented in rodents and zebrafish exposed to 6-OHDA [190,193,217,252,318,319,320,321,322]. While rarely, if ever, used in NHP, rotenone reproduces key features of early and late clinical PD in rodents and zebrafish, with the presence of motor defects, DA loss, and αSyn inclusions in nigral neurons, and LBs [162,189,191,192,197,205,282,283,285,288,323,324,325,326]. Bradykinesia, rigidity, stooped posture, and mild postural instability are reached with the acute dosing (high dosage, short duration) of MPTP in mice and NHP, accompanied by a rapid degeneration of the DOPAn [198,205,284]. Chronic challenge with lower doses of MPTP results in clinical motor signs and decreased DOPAn numbers in middle-aged NHP [327]. Notably, in acute 6-OHDA and MPTP, and in reserpine models, the initial tyrosine hydroxylase (TH^+^, DA precursor) decrease may revert, suggesting reduced DA production rather that a neuron loss [189,193,197,200,328]. Moreover, acute dosing regimens are seldom accompanied by toxicity potentially leading to death (see Table 2). This strongly suggest that acute dosing regimen do not model well the etiopathogenesis of human PD. This may limit not only the translational relevance of understanding disease mechanisms but also using this knowledge to develop and test therapeutic approaches.

Mechanistically speaking, in addition to their well-known pro-oxidant effect, MPTP and 6-OHDA also produce proteasome inhibition [234], leading to endoplasmic reticulum stress [329] and the upregulation of chaperones involved in cell death [330]. Proteasome inhibition is strongly connected with the concept of protein misfolding in PD, and proteasome inhibitors lead to DOPAn loss, motor abnormalities, and αSyn accumulation in vivo [331]. Small inclusion bodies were also reported in c. elegans, rodents, minipigs, and NHPs exposed to, e.g., 6-OHDA and proteosome inhibitor, but the pathogenic role of αSyn in these models has not been determined. In vitro, proteasome inhibition leads to intracellular accumulation of p52, a pro-apoptotic mediator [332,333]. In line with this, proteasome inhibitors can induce the mitochondrial apoptotic signaling cascade, with the increased release of cytochrome c, ROS, and Bcl-2-associated X (BAX) and activation of caspase 3 and caspase 9 [334,335]. Notably, apoptosis is induced by rotenone and 6-OHDA but not by MPTP in the MN9D cell line and primary cultures [232,233,280], highlighting how the chemical used to induce PD may affect the results and their translation to the clinic. Both proteasome inhibitors (specifically actacystin) and rotenone impair mitochondrial function and reduce intracellular ATP [280,336], which might impair vesicular trafficking and mis/unfolded protein clearance, leading to αSyn accumulation [337]. In a cellular model of rotenone-induced PD, transcriptomic sequencing revealed PTEN-induced putative kinase 1 (PINK1) as one of the most altered pathways in mitophagy, stressing its potential contribution in this specific disease mechanism [291].

Notably, in animal models of early PD phase that recapitulates the presence of αSyn, reserpine, 6-OHDA (independently from the dosage and site of injection) and MPTP (at least under acute schemes) do not result in LBs despite the presence of motor abnormalities [114,189,193,197,198,200,205,208,284,328]. Multiple injections (across 8 weeks) of paraquat lead to 50% reduction in TH^+^ cells and αSyn presence but with the absence of LBs in rodents [53,55,156]. Even genetic models largely fail in this aim (see Table 2), except for the SNCA gene coding for αSyn. Altogether, these results may be interpreted in two ways as follows: (1) as a failure of the model, or (2) as an indication that αSyn is not causative for PD symptoms, as discussed in Section 2.1. Supporting criticisms of a mechanistic role of αSyn and LBs in clinical PD is the fact that at moderate/chronic doses of MPTP leads to αSyn aggregation in mice but not DOPAn loss or motor symptoms [198]. Unilateral injection of αSyn preformed fibrils into mouse striatum leads to neuroinflammation preceding accumulation of pathologic form of αSyn, and thereafter neurodegeneration and motor deficits [310] (Table 3). This highlights the importance of the experimental setting and analysis (e.g., αSyn gene expression, physiologic protein, and fibrils) in elucidating translational pathologic mechanisms [54,55]. Interestingly, the uninjected contralateral striatum displays similar pathophysiological effects to the side injected with αSyn preformed fibrils [310]. This has been interpreted as reflecting a pivotal role for inflammatory mediators in initiating the disease (see also the risk phase; 2.1.3: clinic; and 3.3: models) [53,189,193,310,338,339]. Of relevance for the review, there is a general lack of models exploring inflammatory pathways during the early clinical PD stage (Table 3 and Table 4). Some in vitro–ex vivo models (e.g., cell lines, induced pluripotent stem cells—iPSCs, organoids, and organotypic brain cultures—OBCs) have been used to address specific mechanisms of PD. In SH-SY5Y cells exposed to 6-OHDA, the upregulation of 12 necroptosis-related genes belonging to the inflammatory cascade previously identified in patients with PD has been reproduced [235]. In agreement with the inflammatory hypotheses, in PC12 cells, 6-OHDA initiates a physical interaction among the NEDD4 E3 ubiquitin ligase and the pro-apoptotic regulator RTP801 [340]. A decrease in NEDD4 is responsible for increased RTP801 gene expression, which contributes to neuroinflammation, memory impairment, and mortality in Alzheimer’s disease (AD) [341]. The NEDD4-RTP801 complex has been localized at the synapse level in primary cortical neurons and in TH^+^ neurons from PD patients [340]. Moreover, the lncRNA (long non-coding RNA) Metastasis Associated Lung Adenocarcinoma Transcript 1 (MALAT1) induces inflammatory response in microglia BV2 cells, promoting down regulation of miR-23b-3p (micro RNA-23b-3p), reduced autophagy, increased αSyn formation, and apoptosis in dopaminergic neuronal MN9D cells exposed to rotenone [290,294]. This is not a unique example of epigenetic regulation in PD. A reduction in αSyn accumulation in vitro may be obtained by silencing the lncRNA small nucleolar RNA host gene 14 (SNHG14)/miR-133b [293], the circular RNA circ-Pank1 [295], and NADPH oxidase 4 (NOX4) gene [268], suggesting potential translational alternative therapeutic approaches to PD. Similarly, modulating histone deacetylase 4 (HDAC4), which is upregulated at both a transcript and protein level in PD, may increase cell survival and reduce αSyn levels in cell lines exposed to rotenone [292].

Supportive of the interconnection between epigenetic, redox status, and αSyn accumulation [268,293,295], the lncRNA HOXA transcript antisense RNA, myeloid-specific 1 (HOTAIRM1) is upregulated in PD patients. Its silencing induces HMOX1, a redox sensor and activator of different anti-oxidant genes, preventing apoptosis [264]. In line with this, sorting nexin 5 (SNX5), endosomal trafficking regulator, and ferroptosis are also up regulated, and its knockout reverses GSH/GSSG depletion, ROS increase, and cell death in PC-12 cell and rat model induced by 6-OHDA [229]. Ferroptosis-linked cell death has also been reported in SH-SY5Y exposed to MPTP and paraquat, and PC12 cells challenged MPTP and 6-OHDA [230,270], with cell viability completely restored by deferoxamine (DFO), a ferroptosis inhibitor, and partly restored by the overexpression of the ferritin heavy chain 1 (FTH1) [230]. Ferroptosis, redox stress, protein misfolding [342], and αSyn aggregation are connected [230,270,277,342], but the timeline of the events requires experimental confirmation. Of relevance, iron is one of the anabolites of HMOX1 activity, and hyperactivation of HMOX1 is believed to enhance neurodegeneration by increasing oxidative stress [88,91,92,94,149]. As a matter of notice, bilirubin is another anabolic product of HMOX1 activity, with a speculated opposite (protective) action in DOPAn survival [68,297]. Thus, the use of HMOX1 as a marker of PD is supported by the literature, even if the exact role must be elucidated. Also, the excessive release of DA into the cytoplasm or extracellular space may enhance the pro-oxidant status of the milieu. DA can undergo oxidation, enhancing redox stress, as demonstrated in vitro by Li et al. through the blocking of vesicular monoamine transporter 2 (VMAT2) with reserpine in SH-SY5Y cells [155]. Notably this is one of the few studies performed in a time course approach, thus overlapping prodromal (10% cell death at 12 h) and early clinical PD (50% cell death at 24 h—Table 4). The majority of analyses have been performed only at 24 h, when autophagy and αSyn levels were already significantly increased [155]. This highlights the limited number of studies that focus on understanding the early molecular mechanisms of DOPAn loss. As a basis for this review, we have a solid technical background to mimic the prediagnostic stage both in vitro and in vivo models thus the gap can be filled. Iron-induced redox stress is responsible for lipid peroxidation [277], and it is potentially related to the changes in the level of saturation and length of the phospholipid chains of fatty acids present in PD brain tissue that presumably contribute to αSyn accumulation [343]. Of relevance in modeling PD, a mitochondrial oxidative phosphorylation metabolism prevails at low MPTP dosage (30 μM, 90% cell viability), and a glycolytic metabolism prevails at high MPTP challenge in SH-SY5Y cells (300 μM, 70% of cell viability) [344,345].

**Table 3:** Mechanistic findings in pre-clinical models.

	DA Loss/Involvement	Non-DA Loss/Involvement	Motor Deficits	Non-Motor Deficits	αSyn	LBs	Inflammation	Mitochondria and Redox	Ref. and Short Description
Post-diagnosis phase	Y	Y	Y	Y	Y	°	Y	°	[241] Mice, MPTP
	Y	Y	Y	Y	Y	°	Y	Y	[242] Mice, MPTP
	Y	Y	Y	°	°	°	Y	Y	[298] Rat, LPS
	Y	Y	Y	°	°	°	Y	°	[299] Rat, LPS
	°	Y	Y	Y	°	°	°	Y	[300,301] Rat, LPS
	Y	Y	Y	°	Y	°	Y	°	[302] Mice, LPS
	°	Y	Y	°	Y	No	Y	°	[261,262] NHP, MPTP
	Y	°	Y	Y	°	°	Y	°	[207] Mice, 6-OHDA
	Y	°	Y	°	°	°	°	Y	[209] Rat, 6-OHDA
	Y	°	Y	°	°	°	Y	°	[219] Mice, 6-OHDA *
	Y	°	Y	°	°	°	Y	°	[243] Mice, MPTP
	Y	°	Y	°	°	°	°	Y	[210] Mice, 6-OHDA
	Y	°	°	°	°	°	Y	°	[220] Rat, 6-OHDA *
	Y	°	°	°	°	°	°	Y	[221] Zebrafish, 6-OHDA
	Y	°	°	°	°	°	Y	Y	[257] Mice, MPTP *
	Y	°	°	°	°	°	Y	°	[258] Mice, MPTP
	Y	°	°	°	°	°	Y	°	[238] Mice, MPTP
	Y	°	Y	°	°	°	°	Y	[244] Mice, MPTP *
	Y	°	Y	°	°	°	°	Y	[245] Zebrafish, MPTP *
	Y	°	Y	°	°	°	°	Y	[286] Rat, rotenone
	Y	°	Y	°	°	°	Y	°	[283] Mice, rotenone
	Y	°	Y	°	°	°	°	Y	[271] Rat, paraquat
	Y	°	Y	°	°	°	Y	°	[303] Rat, LPS
	Y	°	Y	GI	°	°	Y	°	[307] Rat, LPS
	Y	°	°	°	Y	°	Y	°	[54] Rat, αSyn *
	Y	Y	Y	Y	°	°	°	°	[211] Rat, 6-OHDA *
	Y	Y	Y	Y	°	°	°	°	[212] Rat, 6-OHDA *
	Y	Y	Y	Y	°	°	°	°	[213] Rat, 6-OHDA *
	Y	Y	Y	°	°	°	°	°	[214] Zebrafish, 6-OHDA or MPTP *
	Y	°	Y	°	°	°	°	°	[246] NHP, MPTP *
	Y	Y	Y	°	°	°	°	°	[247] NHP, MPTP
	Y	Y	Y	°	°	°	°	°	[248] NHP, MPTP
	Y	No	Y	No	No	No	°	°	[249] NHP, MPTP *
	Y	Y	Y	Y	°	°	°	°	[250] Mice, MPTP *
	Y	°	Y	°	°	°	°	°	[251] Zebrafish, MPTP
	Y	Y	Y	°	°	°	°	°	[252] Zebrafish, MPTP
	°	°	Y	°	°	°	°	°	[253] Zebrafish, MPTP
	Y	°	Y	°	Y	°	°	°	[285] Rat, rotenone
	Y	°	Y	°	°	°	°	°	[287] Rat, rotenone
	Y	°	Y	Y	°	°	°	°	[272] Zebrafish, paraquat
	Y	°	Y	°	°	°	°	°	[273] Rat, paraquat *
Prodromal to post-diagnosis	Y	°	Y	°	°	°	Y	°	[346] DJ1 mice genetic model *
	Y	°	Y	°	°	°	°	°	[246] NHP, MPTP *
	Y	°	Y	°	°	°	°	°	[200] Mice, reserpine *
	Y	°	Y	°	°	°	Y	°	[346] Park2 mice genetic model
	Y	°	Y	°	No	No	Y	°	[347] Parkin rat genetic model *
	Y	°	Y	°	Y	Y	Y	°	[310] Mice, αSyn *
	Y	°	°	°	°	°	°	°	[222] Rat, 6-OHDA *
	Y	°	Y	°	Y	°	°	°	[311] Mice, LPS and αSyn *
	°	°	Y	°	°	°	Y	°	[67] Rat, LPS
	Y	°	Y	°	°	°	Y	°	[304] Mice, LPS *
	Y	Y	Y	°	°	°	°	Y	[274] Zebrafish, paraquat *
	Y	Y	Y	°	Y	°	Y	Y	[305] Rat, LPS *
	Y	°	Y	°	Y	Y	Y	°	[288] Mice, rotenone *
	Y	°	Y	°	°	°	Y	Y	[289] Mice, rotenone *
	Y	°	Y	°	Y	°	Y	°	[282] Rat, rotenone *
	Y	°	Y	°	°	°	Y	°	[254] NHP, MPTP
	Y	°	Y	°	°	°	°	°	[215] Rat, 6-OHDA
	Y	Y	Y	°	°	°	°	°	[216] Rat, 6-OHDA
	Y	°	Y	Y	°	°	°	°	[255] Rat, 6-OHDA * [255] Rat, MPTP *
	Y	Y	Y	Y	°	°	°	°	
	Y	°	Y	°	weak	°	Y	°	[312] Mice, αSyn
	Y	°	Y	°	°	°	Y	°	[217] Zebrafish, 6-OHDA *
Prodromal phase	Y	No	Y	Y	°	°	°	°	[218] Rat, 6-OHDA
	°	°	°	Y	°	°	°	Y	[223] Rat, 6-OHDA
	Y	°	Y	Y	°	°	°	°	[256] NHP, MPTP
Risk phase	Y	°	°	°	°	°	Y	Y	[306] Rat, LPS *
Out of classification based on the clinical cardinal symptoms	Y	°	°	°	Y	Y	Y	°	[348] αSyn mice genetic model, LPS
	°	°	°	°	°	°	Y	Y	[269] Mice, MPTP *
	Y	°	°	°	Y	°	°	°	[309] Mice, LPS *
	Y	°	°	°	°	°	Y	Y	[308] Mice, LPS

°: not assessed; Y: yes present; No: not present. Abbreviations: LBs: Lewy bodies; DA: dopamine; αSyn: α-synuclein; 6-OHDA: 6-hydroxydopamine; MPTP: 1-methyl-4-phenyl-1,2,3,6-tetrahydropyridine; LPS: lipopolysaccharide; NHP: non-human primates; GI: gastro intestinal. * Models investigating changes along a time course experimental scheme.

#### 3.1.2. Modeling the Late Stages of Clinical PD

As shown in Figure 1 in the late PD stage both motor and non-motor symptoms are present. Behaviors modeling anxiety, depression, and apathy, as well as aphagia, adipsia, and death can result from 6-OHDA and MPTP administration, with a consistent neuron loss (NE loss up to 75%; DA 83–98%) in rodents with intracisternal injection [156,160,189,193,318]. Parallel effects are observed in zebrafish [190,318,324,349,350]. Non-motor PD signs [351], NMDA-linked parkinsonian symptoms [248,352], and NE depletion [247,351] result from chronic treatments with MPTP in NHPs. The degeneration of nigrostriatal DOPAn results in increased GABAergic activity in the striatum [353]. In OBCs from the nucleus of Meynert, cortex, ventral mesencephalon, and dorsal striatum, rotenone treatment results in cholinergic cell death [354]. Loss of SE, Chol, and NE neurons [55,254], with consequent non-motor signs of PD [160,191,324,355], including gastrointestinal dysfunctions, have been documented [53,116,162,191,192,198,283,288,323]. A recent study revealed that MPP^+^-induced effects on DA release are mediated in part by mechanisms related to acetylcholine regulation [356]. The use of rotenone into rodents has been limited by variability in the percentage of animals affected, the long time required for chronic dosing, and high mortality with acute dosing [192]. These limitations have been overcome by using middle-aged rats and medium-chain triglyceride Miglyol 812 N as adjuvant. This regimen results in PD signs in 100% of animals within 3 weeks and minimal inter-individual variability of disease severity [195,285]. Given the completeness of phenotypic signs and the homogeneity in their development, this is likely one of the most effective models currently available for performing time course evaluations of biomolecular events ongoing in the interconnected CNS circuits. Moreover, it also provides valuable insights into disease onset and progression, with minimal ethical and economic investments.

**Table 4:** Mechanistic findings in in vitro and ex vivo models.

	Inflammation	Redox Stress	Autophagy, lysosome, αSyn	Apoptosis	Ref. and Short Description
Post-diagnosis phase (cell death of at least 50%)	°	°	°	°	[357] OBCs, reserpine
	°	Y	Y	Y	[155] SH-SY5Y, reserpine
	°	°	°	Y	[358] OBCs, 6-OHDA
	°	°	Y ^§^	°	[343] SH-SY5Y, 6-OHDA ^§^
	°	°	°	Y	[359] SH-SY5Y, 6-OHDA
	°	Y	°	Y	[225] SH-SY5Y, 6-OHDA
	°	°	°	Y	[359] PC12, 6-OHDA
	°	Y	°	Y	[229] PC12, 6-OHDA
	°	Y	°	°	[230] PC12, 6-OHDA
	°	°	°	Y	[360] LUHMES, 6-OHDA
	°	Y	°	Y	[276] SH-SY5Y, paraquat and physical stretch
	°	Y	°	Y	[277] SH-SY5Y, paraquat
	°	°	°	°	[361] SH-SY5Y, paraquat
	°	Y	°	Y	[263] SH-SY5Y, paraquat *
	°	°	°	Y	[354] OBCs, rotenone
	°	Y	Y	Y	[293] MN9D, rotenone
	Y	°	Y	Y	[294] MN9D, rotenone
	°	°	Y	Y	[295] MN9D, rotenone
	Y	°	Y	Y	[290] LUHMES, rotenone
	°	Y	°	Y	[266] PC12, rotenone
	°	°	Y	°	[330] SH-SY5Y, MPTP
	°	Y	°	Y	[264] SH-SY5Y, MPTP
	°	°	°	Y	[360] SH-SY5Y, MPTP
	°	Y	Y	Y	[362] SH-SY5Y, MPTP
	°	Y	°	Y	[265] SH-SY5Y, MPTP *
	°	Y	°	Y	[266] PC12, MPTP
	Y	Y	°	Y	[363] PC12, MPTP
	°	Y	°	Y	[270] PC12, MPTP
	Y	Y	°	Y	[267] MN9D, MPTP
	°	°	°	Y	[364] MN9D, MPTP
	°	Y	°	Y	[268] MN9D, MPTP
Prodromal to post-diagnosis phase	Y	Y	°	Y	[297] OBCs, rotenone *
	Y	Y	°	Y	[154] OBCs, rotenone *
	°	Y	°	Y	[263] SH-SY5Y, rotenone *^,#^
	°	Y	°	Y	[263] SH-SY5Y, MPTP *^,#^
	°	°	°	Y	[280] MN9D, rotenone ^#^
	°	°	°	°	[345] SH-SY5Y, MPTP
Out of classification based on the clinical cardinal symptoms (no data on cell viability, studied only the molecular mechanisms)	°	°	Y	°	[202] PC12, reserpine
	°	Y	°	°	[201] PC12, reserpine
	°	Y	°	°	[227] SH-SY5Y, 6-OHDA *
	°	Y	°	Y	[228] SH-SY5Y, 6-OHDA
	Y	°	°	°	[235] SH-SY5Y, 6-OHDA
	°	°	°	Y	[340] PC12, 6-OHDA
	°	Y	°	Y	[226] MN9D, 6-OHDA
	°	°	°	Y	[232] MN9D, 6-OHDA
	°	°	Y	°	[234] MN9D, 6-OHDA
	°	Y	°	Y	[233] MN9D, 6-OHDA *
	°	Y	°	Y	[231] MN9D, 6-OHDA
	°	°	°	°	[365] LUHMES, 6-OHDA
	°	Y	°	Y	[275] OBCs, paraquat
	°	°	Y	Y	[292] SH-SY5Y, rotenone
	Y	Y	°	Y	[366] SH-SY5Y, rotenone
	°	°	Y	Y	[296] PC12, rotenone
	°	°	°	°	[329] PC12, rotenone
	°	°	°	°	[153] OBCs, MPTP
	°	°	°	°	[356] OBCs, MPTP
	°	Y	°	Y	[367] SH-SY5Y, MPTP
	°	°	°	°	[329] PC12, MPTP
	°	Y	Y	Y	[368] PC12, MPTP
	°	°	Y	°	[234] MN9D, MPTP

^§^ supposingly linked to αSyn quantity; ^#^ depend on time and concentration of the treatment; * models investigating changes along a time course experimental scheme. Abbreviations: Y: assessed; °: not assessed; αSyn: α-synuclein; 6-OHDA: 6-hydroxydopamine; MPTP: 1-Methyl-4-phenylpyridinium; OBCs: organotypic brain culture; LUHMES: Lund human mesencephalic cell line; MN9D: mouse dopaminergic neuronal cell line.

### 3.2. Modeling the Prodromal Phase

The constellation of non-motor symptoms that are present during the prodromal phase of PD (Figure 1) provide opportunities to establish an early therapeutic window. These symptoms result from the pathology in DA and non-DA pathways in brain regions outside the nigrostriatal pathway (Figure 2). Both the pre-diagnosis symptoms, as well as NA, SE, and Chol dysfunction in extra nigrostriatal areas have been reproduced by in vivo models (6-OHDA; MPTP; paraquat; rotenone; reserpine; and VMAT2, LRRK2, and hA53T αSyn models, see Table 2) [156,157,158,159,160,161,162,163]. For example, hyposmia results from the intranasal infusion of a low MPTP dose in rats [369]. Accompanying hyposmia were TH^+^ decreases in the olfactory bulb, SN, striatum, and prefrontal cortex, followed by cognitive and motor deficits. This model is interesting because these effects recapitulate the prodromal stage, the symptomatic PD stage, and the risk phase as they result from an environmental toxin entering the brain through the olfactory tract. Similar results have been reported following intranigral stereotaxic injection of rotenone in rats. In the model, deceased DA in the SN and hyposmia are correlated with rapid eye movement (REM) sleep deficits [370]. Sleep disturbances are also produced also by 6-OHDA in rodents [224] and NHP [224,259,260]. Ex vivo (OBCs) VMAT2 blockage with reserpine inhibits DA and glutamatergic synaptic activity in the prefrontal cortex, hippocampus, and VTA, key cognitive and affective nuclei involved in prodromal and late PD symptoms. Applying DA restored synaptic activity early during the reserpine challenge but not later on [357], in agreement with L-DOPA therapy in the clinical setting. By extensively reproducing clinical data in prodromal PD, OBCs have shown a decreased synaptic spine number when treated with MPTP [153] and decreased neurite length and number when treated with rotenone [154]. This mimics the decreased synaptic DA preceding DOPAn loss noticed in clinic by 18-F Dopa scan/PET scan, thus supporting the utility of these ex vivo slow degenerating models in studying prodromal mechanisms of PD. In PC12 cells exposed to reserpine, the depletion of intracellular DA results in increased amounts of intracellular GSH, while GSH depletion results in DA demise, supporting the protective anti-oxidant role of GSH on DA, not only in the extracellular matrix, but also intracellularly [201].

Although rarely studied, the blood–brain barrier (BBB) may play a role in PD. As reported in the Lund Human Mesencephalic (LUHMES) cell model with a mild lesion induced by 6-OHDA and MPTP, damage may be reversed by exposing the cells to a pericyte-conditioned medium, emphasizing the importance of cells surrounding the BBB in the initial stages of the disease [360].

Gastrointestinal disorders (GI in Table 2), such as constipation and reduced intestinal motility, both of which are thought to precede classical signs of PD in humans, have been reported in rodents and primates exposed to chronic dosages of MPTP and rotenone [164,165,166]. Notably, the enteric DA level is also altered in these models. Enteric DA plays an immunomodulatory role [68], so the PD-induced DA modulation supports an additional link between PD and inflammation. Considering the putative role of the gastroenteric system in the risk-phase of PD, the causative dynamic among gastrointestinal disorders, DA loss, inflammation, and signs of PD should receive more attention.

### 3.3. Modeling the Risk Phase

Intragastric rotenone has been used to test (with positive results) the Braak hypotheses that PD synucleinopathy starts in the enteric system [371] and reaches the brain through the vagus nerve where it propagates like a prion [35,38,372]. Olfactory dysfunction is a well-known symptom of PD, occurring in 90%–96% of patients [373,374]. The absence of suitable models limits the study of olfactory pathology to post mortem samples from PD patients. Nevertheless, in organotypic olfactory bulb cultures, monoamine-induced DA loss occurs from the prediagnostic to prodromal phases of PD. This is mainly due to oxidative stress, mitochondrial dysfunction, and transcriptomic alterations [375]. The alterations include the upregulation of the amyloid-beta precursor protein (APP), hyperexpression of BDNF, and dysregulation of PARK genes such as PINK1 and DJ-1/PARK7, key factors involved in mitochondrial functioning and oxidative stress regulation [375].

Multiple genetic models of PD exist, frequently failing to reproduce all the classic clinical signs (Table 2) [314,376]. This supports the idea that only a minority of PD cases have a purely genetic origin. In short, mutations in DJ1/PARK7 belonging to the anti-oxidant defense system, lead to a weak PD phenotype without DA loss [193]. Supportive is the fact that the DJ1 genetic model in zebrafish has behavioral deficits, but only in the mitochondrial dysfunction among the cardinal molecular signs of PD [318,377]. In line with the involvement of DJ1 in redox-mediated disease processes, rodents with DJ1 KO challenged with 6-OHDA exhibited greater DA depletion, loss of TH^+^ neurons, and worse performance in behavioral tests compared to WT mice treated with 6-OHDA [378]. Considering the interplay among genes and environment, these findings support this mutation as a risk factor associated with redox stress [379], a condition that is prevalent in many neurodegenerative diseases. As a part of the PINK1–PARKIN protein complex that is required for the removal of damaged mitochondria, their malfunctioning leads to secondary absence of autophagy and accumulation of αSyn [379]. Specifically, the loss of the E3 ubiquitin ligase PARKIN leads to proteasomal dysfunctions and to the accumulation of parkin substrates (e.g., synphilin-1, O-glycosylated alpha-synuclein, Pael-R, CHIP, cdc-Rel1A, cyclin E, and synaptotagmin X1) [380]. Despite this, the genetic models do not reproduce LB formation and neurodegeneration [189,376]. Again, only the PARKIN-Q311X-DAT-BAC mice manifest progressive motor deficits and loss of nigrostriatal DA neurons. This mutation also results in dose-dependent neurodegeneration in rats [347]. Effects of PINK1 mutation in zebrafish is limited to behavioral deficits and mitochondrial dysfunction [318]. These models exhibit a lack of DOPAn loss in SN, mild locomotor disabilities [189,376], and mild anxiety behaviors [160,197]. PARKIN deficiency may also enhance the vulnerability of SN neurons to inflammation [381]. Supporting this was a study reporting that when PARKIN^+/−^ were injected with LPS, an inflammasome inhibitor reduced inflammation, neurodegenerative damage, and increased TH^+^ rescue compared to WT animals undergoing the same treatment. This highlights the role of PARKIN in modulating the inflammasome [382]. αSyn (SNCA) models often do not present DA loss, and most studies do not include behavioral testing, instead focusing on the measures of inflammation and microglia activation [189,193,197,205]. The zebrafish SNCA model exhibits reduced DA, mitochondrial activity, and motor deficits [318,383,384,385]. As discussed in the clinical section, the interplay among genetic, environmental, and lifestyle factors is now considered the most likely responsible for PD onset. In line, in a rodent αSyn transgenic model challenged with LPS, LPS triggered a progressive degeneration of the nigrostriatal dopamine pathway, αSyn aggregation and LBs formation, not present in the pure genetic model [348]. Interestingly, rotenone administration in genetic models of αSyn recapitulate the constellation of symptoms present in human PD. This is not possible using a single inducer, suggesting that αSyn overexpression enhances DA sensitivity to mitochondrial impairment and oxidative stress [191]. Nevertheless, as for the other PD-linked mutations, iPSCs derived from patients with SNCA variants are powerful tools for developing personalized medicine approaches [316,386]. Generally, mutations in LRRK-2 lead to mitochondrial dysfunction, oxidative stress, inflammation, autophagy, and proteasomal dysfunction [161], but not αSyn and LB formation, neurodegeneration, or frank motor deficits in animal models [55,161,189,197]. DOPAn loss in SN is present only with the G2019S LRRK2 mutation in rodents [387]. This mutation in iPSCs increases cell death and reduces branching complexity in DOPAn [388]. Like αSyn, zebrafish with LRRK2 mutation exhibit a more complete classic PD phenotype, with neuronal loss, αSyn aggregation, and motor deficits [318]. In iPSCs cells from a patient with LRRK2 variants and early on-set PD, increased expression of different oxidative stress-related genes and αSyn protein were detected [317].

Inflammation is gaining recognition as a potential trigger of clinical PD, and emerging studies are beginning to explore this hypothesis (Table 3). Choi et al. described in rats a progressive reduction when injected with LPS in the number of TH^+^ cells in SN (21%, 38%, and 41% at 1, 2, and 4 weeks, respectively) compared to the non-injected side [194,305]. These resulting data have been reported in rats [305,389,390] and mice [391], supporting the reproducibility of the model. Of relevance, these studies belong to the minority of experimental studies that measure PD readouts over time (Table 3 and Table 4), providing valuable temporal mechanisms that may be related to PD progression. Even more relevant to the risk phase, intrauterine injection of LPS in pregnant rodents leads not only to decreased DA in the brains of offspring, but also to enhanced damage from pro-inflammatory stimuli administered in the post-natal age [306,392]. In line with this, in OBCs challenged with low doses of rotenone to recreate a slow degenerative model of PD, inflammation, and specifically TNFα, has been demonstrated to be the determinant of DOPAn demise [154,297]. Notably, TNFα overexpression in SN leads to progressive neurodegeneration, with motor signs and microgliosis, demonstrating the pivotal role of this cytokine in DA loss [67]. Along risk factors, low systemic inflammation is present in diabetes, and in PC12 cells exposed to 6-OHDA cell death is almost completely reversed by the silencing of insulin growth factor binding protein 5 (IGFBP5) [359]. These results have been confirmed in vivo, where wild-type (WT) rats exposed to 6-OHDA presented motor abnormalities and TH^+^ decrease, while not observed in IGFBP5^−/−^ animals [359]. In line with this, in aging studies, IGFBP5 is correlated with cognitive dysfunction, also present in PD [393]. Aging, obesity, pre-diabetes, type 2 diabetes, and insulin resistance are debated risk factors in human PD, as discussed by others [33,172,177,394,395,396,397,398]. Conversely, low-dose LPS preconditioning to midbrain OBCs protected DOPAn against inflammatory damage by decreasing microglial activation and releasing pro-inflammatory factors [399]. A complementary in vitro approach (co-culture of SH-SY5Y cells, BV2 cells, and primary microglia) suggested that the adaptative anti-inflammatory behavior results from suppressing the TLR4-mediated NF-κB and MAPK p38 signaling pathways [399]. This suggests that the level of inflammation (and possibly TNFα) may determine the protective or damaging sequence in PD.

## 4. Conclusions

Due to the complexity of Parkinson’s disease (PD), current clinical management is largely limited to symptom control. There is a significant lack of prevention campaigns and disease-modifying strategies. To address these gaps, existing PD models should be more extensively and strategically utilized (Box 1). Clinical knowledge should serve as a validation tool for assessing the reproducibility of newly developed models, and it should not be the final goal. A critical need exists to accurately recapitulate the chronic progression of PD, especially to investigate the pre-diagnostic stages to identify biomolecular changes in the early phases. Our review highlights a striking lack of studies from risk to pre-clinical stages.

Box 1 **Key-notes regarding the main failures/limits of the current models.**

- **Acute schemes approach does not model consistently the etiopathogenesis of human PD**. This limits the translational relevance in the understanding of the disease and therapies screening.
- **Focus on synucleopathy**. The role of synucleopathy is debated, possibly relevant only to a subtype of PD. Possibly a late event.
- **Up to now, large attention to the late (after diagnosis) molecular events**. Need for discovery approaches, necessity in exploring the early stages.
- **Disease complexity**. Multiple mechanisms and neurotransmitters involved, in a different time scale. Need for experimental schemes exploring the different phases in a single study.
- **Need for complex models**. The temporal scale of human PD is too long for experimental studies. Models are necessary and fundamental.
- **Need for solid, reproducible and shared models**. The past studies build the basis on knowledge in how to properly mimic human PD. Using this background to pursuit the objectives suggested in the previous points by creating slow degenerative models, by using shared, uniform protocols, making data comparable and potentially complementary might be a plus.

Researchers often struggle to select the most appropriate model, as the predominant focus remains on the clinical (symptomatic) phase of PD. Research often isolates individual mechanisms without integrating them into the broader, interconnected reality of PD. Current knowledge demonstrates the extreme complexity of PD. Multiple and interconnected neurotransmitter systems are involved. Many non-motor symptoms described are present both in the prodromal and late phase. The extra CNS origin is more and more believed, not only the Braak hypotheses, but the interplay of genes with inflammation, as an example. The most promising approach to fill the gap looks to be the use of the diverse range of PD models with milder disease triggers replicating the early stages, both in vitro and in vivo. The study of multiple experimental times, in slow degenerative models, by a discovery approach will be ideal. In vitro models remain crucial for dissecting intrinsic cellular signaling pathways. Genetic models, although not fully representative of all PD symptoms or outcomes, are invaluable for studying the pathological consequences of specific mutations and the interaction between genomic variants, the environment, and lifestyle. Continuing developing and using complex in vivo models, despite the ethical concerns they raise, is fundamental to overstep the limitation of specimens, excessively long timing, and the ethical concerns of clinical studies on PD patients.

A comprehensive view that balances complexity with clinical applicability and reproducibility is essential. Despite its high public profile, PD biologically remains largely enigmatic. Clinical and, above all, basic research must continue to delve deeper into its mechanisms, exploiting new omics technologies to perform discovery of the disease.

## Data Availability

All the data are included in the manuscript.

## Abbreviations

The following abbreviations are used in this manuscript:

5-HT 2A, 1A	serotonin 2A, 1A receptor
6-OHDA	6-Hydroxydopamine
ADA	adenosine 2A receptor
AMP	cyclic adenosine monophosphate
AMPA	α-amino-3-hydroxy-5-methyl-4-isoxazolepropionic acid receptor
αSyn	αSynuclein
ATP	Adenosine triphosphate
BAX	Bcl-2 Associated X
BBB	blood–brain barrier
BDNF	brain-derived neurotrophic factor
Chol	choline/cholinergic
CNS	central nervous system
COX	cyclooxygenase
CSF	cerebrospinal fluid
CXCL12	cytokine C-X-C motif chemokine ligand 12
D1, 2	dopamine receptor 1, 2
DA	dopamine
DAT	dopamine transporter
DAT SPECT	dopamine transporter single-photon emission computed tomography
DJ1	deglycase J1 (PARK7)
DOPAn	dopaminergic neurons
FTH1	ferritin heavy chain 1
GABA	gamma-aminobutyric acid receptor
GDNF	glial-derived neurotrophic factor
GI	gastrointestinal
Glut	glutamate
GPe	external globus pallidus
Gpi	internal globus pallidus
GSH	reduced glutathione
H&Y	Hoehn & Yahr score
HDAC4	histone deacetylase 4
HMOX1	heme oxygenase 1
HOTAIRM1	HOXA transcript antisense RNA, myeloid-specific 1
IFNγ	interferon-γ
IGFBP5	insulin growth factor binding protein 5
IL	interleukin
LBs	Lewy body
lncRNA	long non-coding RNA
LPS	lipopolysaccharides
LRRK2	leucine-rich repeat kinase 2
M1, 2, 4	muscarinic acetylcholine receptor 1, 2, 4
MALAT1	metastasis associated lung adenocarcinoma transcript 1
MAO-B	glial monoamine oxidase B
MAPK	mitogen-activated protein kinases
mGluR5	metabotropic glutamate receptor 5
MHCII	major histocompatibility complex class II
miR	micro-RNA
MPTP	1-Methyl-4-phenyl-1,2178,3,6-tetrahydropyridine
MPP+	1-methyl-4-phenylpyridinium
MRI	magnetic resonance imaging
nAChR	nicotinic acetylcholine receptors
NADH	nicotinamide adenine dinucleotide-coenzyme 1
NFκB	nuclear factor kappa-light-chain-enhancer of activated B cells
NHPs	non-human primates
NLRP3	NOD-, LRR-, and pyrin domain-containing protein 3
NMDA	N-methyl-D-aspartate receptor
NOS	nitrogen oxygen species
NOX4	NADPH oxidase 4
NRF2	nuclear factor erythroid 2-related factor 2
OBCs	organotypic brain cultures
PD	Parkinson’s disease
PET	positron emission tomography scan
PINK1	PTEN-induced kinase 1
PKA	protein kinase A
PPARs	peroxisome proliferator-activated receptors
PRKN	parkin RBR E3 ubiquitin protein ligase
RBD	REM sleep behavior disorders
ROS	reactive oxygen species
SN	substantia nigra
SNCA	synuclein alpha gene
SNHG14	small nucleolar RNA host gene 14
SNpc	substantia nigra pars compacta
SNpr	substantia nigra pars reticulata
SOD	superoxide dismutase
TLR4	toll-like receptor 4
TNFα	tumor necrosis factor alpha
UCHL1	ubiquitin carboxyl-terminal hydrolase isozyme L1
UPDRS	unified PD rating scale
VMAT2	vesicular monoamine transporter 2
WT	wild-type
α2A, 2C	α2A, 2C adrenergic receptor

## References

[1] Dommershuijsen L.J., Boon A.J.W., Ikram M.K. (2021). Probing the Pre-diagnostic Phase of Parkinson’s Disease in Population-Based Studies. Front. Neurol..

[2] Kalia L.V., Lang A.E. (2015). Parkinson’s disease. Lancet.

[3] Savica R., Rocca W.A., Ahlskog J.E. (2010). When does Parkinson disease start?. Arch. Neurol..

[4] Nieoullon A. (2002). Dopamine and the regulation of cognition and attention. Prog. Neurobiol..

[5] Klingelhoefer L., Reichmann H. (2015). Pathogenesis of Parkinson disease—The gut-brain axis and environmental factors. Nat. Rev. Neurol..

[6] Liang L., DeLong M.R., Papa S.M. (2008). Inversion of Dopamine Responses in Striatal Medium Spiny Neurons and Involuntary Movements. J. Neurosci..

[7] Surmeier D.J., Obeso J.A., Halliday G.M. (2017). Selective neuronal vulnerability in Parkinson disease. Nat. Rev. Neurosci..

[8] Hirano S. (2021). Clinical implications for dopaminergic and functional neuroimage research in cognitive symptoms of Parkinson’s disease. Mol. Med..

[9] Schrag A., Horsfall L., Walters K., Noyce A., Petersen I. (2015). Prediagnostic presentations of Parkinson’s disease in primary care: A case-control study. Lancet Neurol..

[10] Noyce A.J., Lees A.J., Schrag A.-E. (2016). The prediagnostic phase of Parkinson’s disease. J. Neurol. Neurosurg. Psychiatry.

[11] Breen D.P., Lang A.E. (2017). Tracking the course of prodromal Parkinson’s disease. Brain.

[12] Siderowf A., Stern M.B. (2006). Preclinical diagnosis of Parkinson’s disease: Are we there yet?. Curr. Neurol. Neurosci. Rep..

[13] Caproni S., Colosimo C. (2020). Diagnosis and Differential Diagnosis of Parkinson Disease. Clin. Geriatr. Med..

[14] Moretti R., Torre P., Antonello R.M. (2013). Parkinson’s Disease: Behavioural and Cognitive Aspects: Behavioural & Cognitive Aspects.

[15] Mantri S., Morley J.F., Siderowf A.D. (2019). The importance of preclinical diagnostics in Parkinson disease. Parkinsonism Relat. Disord..

[16] Hoehn M.M., Yahr M.D. (1967). Parkinsonism: Onset, progression and mortality. Neurology.

[17] Goetz C.G., Poewe W., Rascol O., Sampaio C., Stebbins G.T., Counsell C., Giladi N., Holloway R.G., Moore C.G., Wenning G.K. (2004). Movement Disorder Society Task Force report on the Hoehn and Yahr staging scale: Status and recommendations The Movement Disorder Society Task Force on rating scales for Parkinson’s disease. Mov. Disord..

[18] Brück A., Aalto S., Nurmi E., Vahlberg T., Bergman J., Rinne J.O. (2006). Striatal subregional 6-[18F]fluoro-L-dopa uptake in early Parkinson’s disease: A two-year follow-up study. Mov. Disord. Off. J. Mov. Disord. Soc..

[19] Greffard S., Verny M., Bonnet A.-M., Beinis J.-Y., Gallinari C., Meaume S., Piette F., Hauw J.-J., Duyckaerts C. (2006). Motor score of the Unified Parkinson Disease Rating Scale as a good predictor of Lewy body-associated neuronal loss in the substantia nigra. Arch. Neurol..

[20] Nandhagopal R., Kuramoto L., Schulzer M., Mak E., Cragg J., Lee C.S., McKenzie J., McCormick S., Samii A., Troiano A. (2009). Longitudinal progression of sporadic Parkinson’s disease: A multi-tracer positron emission tomography study. Brain J. Neurol..

[21] Hawkes C.H. (2008). Parkinson’s disease and aging: Same or different process?. Mov. Disord. Off. J. Mov. Disord. Soc..

[22] Hawkes C.H., Del Tredici K., Braak H. (2010). A timeline for Parkinson’s disease. Parkinsonism Relat. Disord..

[23] Obeso J.A., Rodriguez-Oroz M.C., Goetz C.G., Marin C., Kordower J.H., Rodriguez M., Hirsch E.C., Farrer M., Schapira A.H.V., Halliday G. (2010). Missing pieces in the Parkinson’s disease puzzle. Nat. Med..

[24] Jenner P. (2003). Oxidative stress in Parkinson’s disease. Ann. Neurol..

[25] Moore D.J., West A.B., Dawson V.L., Dawson T.M. (2005). Molecular pathophysiology of parkinson’s disease. Annu. Rev. Neurosci..

[26] Lang A.E. (2007). The progression of Parkinson disease. Neurology.

[27] Cohen G. (2000). Oxidative stress, mitochondrial respiration, and Parkinson’s disease. Ann. N. Y. Acad. Sci..

[28] McNaught K.S.P., Belizaire R., Isacson O., Jenner P., Olanow C.W. (2003). Altered proteasomal function in sporadic Parkinson’s disease. Exp. Neurol..

[29] Hirsch E.C., Hunot S., Damier P., Faucheux B. (1998). Glial cells and inflammation in parkinson’s disease: A role in neurodegeneration?. Ann. Neurol..

[30] Teismann P., Tieu K., Choi D.-K., Wu D.-C., Naini A., Hunot S., Vila M., Jackson-Lewis V., Przedborski S. (2003). Cyclooxygenase-2 is instrumental in Parkinson’s disease neurodegeneration. Proc. Natl. Acad. Sci. USA.

[31] Mattissek C., Teis D. (2014). The role of the endosomal sorting complexes required for transport (ESCRT) in tumorigenesis. Mol. Membr. Biol..

[32] Teismann P., Tieu K., Cohen O., Choi D.-K., Wu D.C., Marks D., Vila M., Jackson-Lewis V., Przedborski S. (2003). Pathogenic role of glial cells in Parkinson’s disease. Mov. Disord. Off. J. Mov. Disord. Soc..

[33] Tansey M.G., Goldberg M.S. (2010). Neuroinflammation in Parkinson’s disease: Its role in neuronal death and implications for therapeutic intervention. Neurobiol. Dis..

[34] Koziorowski D., Figura M., Milanowski Ł.M., Szlufik S., Alster P., Madetko N., Friedman A. (2021). Mechanisms of Neurodegeneration in Various Forms of Parkinsonism-Similarities and Differences. Cells.

[35] Braak H., Braak E. (2000). Pathoanatomy of Parkinson’s disease. J. Neurol..

[36] Braak H., Del Tredici K., Rüb U., de Vos R.A.I., Jansen Steur E.N.H., Braak E. (2003). Staging of brain pathology related to sporadic Parkinson’s disease. Neurobiol. Aging.

[37] Burke R.E., Dauer W.T., Vonsattel J.P.G. (2008). A critical evaluation of the Braak staging scheme for Parkinson’s disease. Ann. Neurol..

[38] Rietdijk C.D., Perez-Pardo P., Garssen J., van Wezel R.J.A., Kraneveld A.D. (2017). Exploring Braak’s Hypothesis of Parkinson’s Disease. Front. Neurol..

[39] Lebouvier T., Neunlist M., Bruley des Varannes S., Coron E., Drouard A., N’Guyen J.-M., Chaumette T., Tasselli M., Paillusson S., Flamand M. (2010). Colonic biopsies to assess the neuropathology of Parkinson’s disease and its relationship with symptoms. PLoS ONE.

[40] Bellomo G., De Luca C.M.G., Paoletti F.P., Gaetani L., Moda F., Parnetti L. (2022). α-Synuclein Seed Amplification Assays for Diagnosing Synucleinopathies: The Way Forward. Neurology.

[41] Waqar S., Khan H., Zulfiqar S.K., Ahmad A. (2023). Skin Biopsy as a Diagnostic Tool for Synucleinopathies. Cureus.

[42] Yulug B., Ozansoy M., Cankaya S. (2019). A different view on the pathophysiology of Parkinson’s disease: A descendent neurochemical hypothesis?. Neural Regen. Res..

[43] Jellinger K.A. (2019). Is Braak staging valid for all types of Parkinson’s disease?. J. Neural Transm. Vienna Austria 1996.

[44] Kalia L.V., Kalia S.K., McLean P.J., Lozano A.M., Lang A.E. (2013). α-Synuclein oligomers and clinical implications for Parkinson disease. Ann. Neurol..

[45] Neupane S., De Cecco E., Aguzzi A. (2023). The Hidden Cell-to-Cell Trail of α-Synuclein Aggregates. J. Mol. Biol..

[46] Mikolaenko I., Pletnikova O., Kawas C.H., O’Brien R., Resnick S.M., Crain B., Troncoso J.C. (2005). Alpha-synuclein lesions in normal aging, Parkinson disease, and Alzheimer disease: Evidence from the Baltimore Longitudinal Study of Aging (BLSA). J. Neuropathol. Exp. Neurol..

[47] Klos K.J., Ahlskog J.E., Josephs K.A., Apaydin H., Parisi J.E., Boeve B.F., DeLucia M.W., Dickson D.W. (2006). Alpha-synuclein pathology in the spinal cords of neurologically asymptomatic aged individuals. Neurology.

[48] Ding Z.-T., Wang Y., Jiang Y.-P., Hashizume Y., Yoshida M., Mimuro M., Inagaki T., Iwase T. (2006). Characteristics of alpha-synucleinopathy in centenarians. Acta Neuropathol..

[49] Parkkinen L., Pirttilä T., Alafuzoff I. (2008). Applicability of current staging/categorization of alpha-synuclein pathology and their clinical relevance. Acta Neuropathol..

[50] Doherty K.M., Silveira-Moriyama L., Parkkinen L., Healy D.G., Farrell M., Mencacci N.E., Ahmed Z., Brett F.M., Hardy J., Quinn N. (2013). Parkin disease: A clinicopathologic entity?. JAMA Neurol..

[51] Bhidayasiri R., Tarsy D., Bhidayasiri R., Tarsy D. (2012). Parkinson’s Disease: Hoehn and Yahr Scale. Movement Disorders: A Video Atlas: A Video Atlas.

[52] Dijkstra A.A., Voorn P., Berendse H.W., Groenewegen H.J., Rozemuller A.J.M., van de Berg W.D.J., Netherlands Brain Bank (2014). Stage-dependent nigral neuronal loss in incidental Lewy body and Parkinson’s disease. Mov. Disord. Off. J. Mov. Disord. Soc..

[53] Dauer W., Przedborski S. (2003). Parkinson’s disease: Mechanisms and models. Neuron.

[54] Harms A.S., Delic V., Thome A.D., Bryant N., Liu Z., Chandra S., Jurkuvenaite A., West A.B. (2017). α-Synuclein fibrils recruit peripheral immune cells in the rat brain prior to neurodegeneration. Acta Neuropathol. Commun..

[55] Blesa J., Phani S., Jackson-Lewis V., Przedborski S. (2012). Classic and new animal models of Parkinson’s disease. J. Biomed. Biotechnol..

[56] Baba M., Nakajo S., Tu P.H., Tomita T., Nakaya K., Lee V.M., Trojanowski J.Q., Iwatsubo T. (1998). Aggregation of alpha-synuclein in Lewy bodies of sporadic Parkinson’s disease and dementia with Lewy bodies. Am. J. Pathol..

[57] Armstrong M.J., Okun M.S. (2020). Diagnosis and Treatment of Parkinson Disease: A Review. JAMA.

[58] Halliday G.M., McCann H. (2010). The progression of pathology in Parkinson’s disease. Ann. N. Y. Acad. Sci..

[59] Selikhova M., Williams D.R., Kempster P.A., Holton J.L., Revesz T., Lees A.J. (2009). A clinico-pathological study of subtypes in Parkinson’s disease. Brain J. Neurol..

[60] Kempster P.A., O’Sullivan S.S., Holton J.L., Revesz T., Lees A.J. (2010). Relationships between age and late progression of Parkinson’s disease: A clinico-pathological study. Brain J. Neurol..

[61] Tansey M.G., Wallings R.L., Houser M.C., Herrick M.K., Keating C.E., Joers V. (2022). Inflammation and immune dysfunction in Parkinson disease. Nat. Rev. Immunol..

[62] Jayanti S., Moretti R., Tiribelli C., Gazzin S. (2021). Bilirubin: A Promising Therapy for Parkinson’s Disease. Int. J. Mol. Sci..

[63] Kannarkat G.T., Boss J.M., Tansey M.G. (2013). The Role of Innate and Adaptive Immunity in Parkinson’s Disease. J. Park. Dis..

[64] Jayanti S., Moretti R., Tiribelli C., Gazzin S. (2020). Bilirubin and inflammation in neurodegenerative and other neurological diseases. Neuroimmunol. Neuroinflamm..

[65] Phani S., Loike J.D., Przedborski S. (2012). Neurodegeneration and inflammation in Parkinson’s disease. Parkinsonism Relat. Disord..

[66] Williams-Gray C.H., Wijeyekoon R., Yarnall A.J., Lawson R.A., Breen D.P., Evans J.R., Cummins G.A., Duncan G.W., Khoo T.K., Burn D.J. (2016). Serum immune markers and disease progression in an incident Parkinson’s disease cohort (ICICLE-PD). Mov. Disord..

[67] De Lella Ezcurra A.L., Chertoff M., Ferrari C., Graciarena M., Pitossi F. (2010). Chronic expression of low levels of tumor necrosis factor-alpha in the substantia nigra elicits progressive neurodegeneration, delayed motor symptoms and microglia/macrophage activation. Neurobiol. Dis..

[68] Jayanti S., Dalla Verde C., Tiribelli C., Gazzin S. (2023). Inflammation, Dopaminergic Brain and Bilirubin. Int. J. Mol. Sci..

[69] Pajares M., I. Rojo A., Manda G., Boscá L., Cuadrado A. (2020). Inflammation in Parkinson’s Disease: Mechanisms and Therapeutic Implications. Cells.

[70] Chen Z., Trapp B.D. (2016). Microglia and neuroprotection. J. Neurochem..

[71] Kouchaki E., Kakhaki R.D., Tamtaji O.R., Dadgostar E., Behnam M., Nikoueinejad H., Akbari H. (2018). Increased serum levels of TNF-α and decreased serum levels of IL-27 in patients with Parkinson disease and their correlation with disease severity. Clin. Neurol. Neurosurg..

[72] Menza M., Dobkin R.D., Marin H., Mark M.H., Gara M., Bienfait K., Dicke A., Kusnekov A. (2010). The role of inflammatory cytokines in cognition and other non-motor symptoms of Parkinson’s disease. Psychosomatics.

[73] Rocha N.P., Teixeira A.L., Scalzo P.L., Barbosa I.G., de Sousa M.S., Morato I.B., Vieira E.L.M., Christo P.P., Palotás A., Reis H.J. (2014). Plasma levels of soluble tumor necrosis factor receptors are associated with cognitive performance in Parkinson’s disease. Mov. Disord. Off. J. Mov. Disord. Soc..

[74] Tansey M.G., McCoy M.K., Frank-Cannon T.C. (2007). Neuroinflammatory mechanisms in Parkinson’s disease: Potential environmental triggers, pathways, and targets for early therapeutic intervention. Exp. Neurol..

[75] Imamura K., Hishikawa N., Sawada M., Nagatsu T., Yoshida M., Hashizume Y. (2003). Distribution of major histocompatibility complex class II-positive microglia and cytokine profile of Parkinson’s disease brains. Acta Neuropathol..

[76] Bosco D.A., Fowler D.M., Zhang Q., Nieva J., Powers E.T., Wentworth P., Lerner R.A., Kelly J.W. (2006). Elevated levels of oxidized cholesterol metabolites in Lewy body disease brains accelerate α-synuclein fibrilization. Nat. Chem. Biol..

[77] Lutters B., Foley P., Koehler P.J. (2018). The centennial lesson of encephalitis lethargica. Neurology.

[78] Limphaibool N., Iwanowski P., Holstad M.J.V., Kobylarek D., Kozubski W. (2019). Infectious Etiologies of Parkinsonism: Pathomechanisms and Clinical Implications. Front. Neurol..

[79] Ascherio A., Schwarzschild M.A. (2016). The epidemiology of Parkinson’s disease: Risk factors and prevention. Lancet Neurol..

[80] Lehmann J.M., Lenhard J.M., Oliver B.B., Ringold G.M., Kliewer S.A. (1997). Peroxisome proliferator-activated receptors alpha and gamma are activated by indomethacin and other non-steroidal anti-inflammatory drugs. J. Biol. Chem..

[81] Jimenez-Ferrer I., Swanberg M. (2018). Immunogenetics of Parkinson’s Disease. Parkinson’s Disease: Pathogenesis and Clinical Aspects [Internet].

[82] Yao L., Wu J., Koc S., Lu G. (2021). Genetic Imaging of Neuroinflammation in Parkinson’s Disease: Recent Advancements. Front. Cell Dev. Biol..

[83] Arena G., Sharma K., Agyeah G., Krüger R., Grünewald A., Fitzgerald J.C. (2022). Neurodegeneration and Neuroinflammation in Parkinson’s Disease: A Self-Sustained Loop. Curr. Neurol. Neurosci. Rep..

[84] Tanner C.M., Kamel F., Ross G.W., Hoppin J.A., Goldman S.M., Korell M., Marras C., Bhudhikanok G.S., Kasten M., Chade A.R. (2011). Rotenone, Paraquat, and Parkinson’s Disease. Environ. Health Perspect..

[85] Schapira A.H., Cooper J.M., Dexter D., Jenner P., Clark J.B., Marsden C.D. (1989). Mitochondrial complex I deficiency in Parkinson’s disease. Lancet.

[86] Wei Z., Li X., Li X., Liu Q., Cheng Y. (2018). Oxidative Stress in Parkinson’s Disease: A Systematic Review and Meta-Analysis. Front. Mol. Neurosci..

[87] Pan T., Kondo S., Le W., Jankovic J. (2008). The role of autophagy-lysosome pathway in neurodegeneration associated with Parkinson’s disease. Brain J. Neurol..

[88] Schipper H.M., Song W., Tavitian A., Cressatti M. (2019). The sinister face of heme oxygenase-1 in brain aging and disease. Prog. Neurobiol..

[89] Yoo M.S., Chun H.S., Son J.J., DeGiorgio L.A., Kim D.J., Peng C., Son J.H. (2003). Oxidative stress regulated genes in nigral dopaminergic neuronal cells: Correlation with the known pathology in Parkinson’s disease. Mol. Brain Res..

[90] Schipper H.M., Liberman A., Stopa E.G. (1998). Neural heme oxygenase-1 expression in idiopathic Parkinson’s disease. Exp. Neurol..

[91] Schipper H.M., Song W., Zukor H., Hascalovici J.R., Zeligman D. (2009). Heme oxygenase-1 and neurodegeneration: Expanding frontiers of engagement. J. Neurochem..

[92] Schipper H.M. (2004). Brain iron deposition and the free radical-mitochondrial theory of ageing. Ageing Res. Rev..

[93] Schipper H.M. (2000). Heme oxygenase-1: Role in brain aging and neurodegeneration. Exp. Gerontol..

[94] Xu J., Xiao C., Song W., Cui X., Pan M., Wang Q., Feng Y., Xu Y. (2021). Elevated Heme Oxygenase-1 Correlates with Increased Brain Iron Deposition Measured by Quantitative Susceptibility Mapping and Decreased Hemoglobin in Patients with Parkinson’s Disease. Front. Aging Neurosci..

[95] Teleanu D.M., Niculescu A.-G., Lungu I.I., Radu C.I., Vladâcenco O., Roza E., Costăchescu B., Grumezescu A.M., Teleanu R.I. (2022). An Overview of Oxidative Stress, Neuroinflammation, and Neurodegenerative Diseases. Int. J. Mol. Sci..

[96] Gao H.-M., Zhou H., Hong J.-S., Peterson P.K., Toborek M. (2014). Oxidative Stress, Neuroinflammation, and Neurodegeneration. Neuroinflammation and Neurodegeneration.

[97] Hely M.A., Reid W.G.J., Adena M.A., Halliday G.M., Morris J.G.L. (2008). The Sydney multicenter study of Parkinson’s disease: The inevitability of dementia at 20 years. Mov. Disord. Off. J. Mov. Disord. Soc..

[98] Broadfoot C.K., Abur D., Hoffmeister J.D., Stepp C.E., Ciucci M.R. (2019). Research-based Updates in Swallowing and Communication Dysfunction in Parkinson Disease: Implications for Evaluation and Management. Perspect. ASHA Spec. Interest Groups.

[99] Buddhala C., Loftin S.K., Kuley B.M., Cairns N.J., Campbell M.C., Perlmutter J.S., Kotzbauer P.T. (2015). Dopaminergic, serotonergic, and noradrenergic deficits in Parkinson disease. Ann. Clin. Transl. Neurol..

[100] Ye Z. (2022). Mapping neuromodulatory systems in Parkinson’s disease: Lessons learned beyond dopamine. Curr. Med..

[101] Agid Y., Cervera P., Hirsch E., Javoy-Agid F., Lehericy S., Raisman R., Ruberg M. (1989). Biochemistry of Parkinson’s disease 28 years later: A critical review. Mov. Disord. Off. J. Mov. Disord. Soc..

[102] Zarow C., Lyness S.A., Mortimer J.A., Chui H.C. (2003). Neuronal loss is greater in the locus coeruleus than nucleus basalis and substantia nigra in Alzheimer and Parkinson diseases. Arch. Neurol..

[103] Hilker R., Thomas A.V., Klein J.C., Weisenbach S., Kalbe E., Burghaus L., Jacobs A.H., Herholz K., Heiss W.D. (2005). Dementia in Parkinson disease: Functional imaging of cholinergic and dopaminergic pathways. Neurology.

[104] Rinne J.O., Ma S.Y., Lee M.S., Collan Y., Röyttä M. (2008). Loss of cholinergic neurons in the pedunculopontine nucleus in Parkinson’s disease is related to disability of the patients. Parkinsonism Relat. Disord..

[105] Thannickal T.C., Lai Y.-Y., Siegel J.M. (2007). Hypocretin (orexin) cell loss in Parkinson’s disease. Brain J. Neurol..

[106] Rodriguez-Oroz M.C., Jahanshahi M., Krack P., Litvan I., Macias R., Bezard E., Obeso J.A. (2009). Initial clinical manifestations of Parkinson’s disease: Features and pathophysiological mechanisms. Lancet Neurol..

[107] Dickson D.W. (2012). Parkinson’s Disease and Parkinsonism: Neuropathology. Cold Spring Harb. Perspect. Med..

[108] Fox S.H. (2013). Non-dopaminergic treatments for motor control in Parkinson’s disease. Drugs.

[109] Gonzalez-Latapi P., Bhowmick S.S., Saranza G., Fox S.H. (2020). Non-Dopaminergic Treatments for Motor Control in Parkinson’s Disease: An Update. CNS Drugs.

[110] Coelho M., Ferreira J.J. (2012). Late-stage Parkinson disease. Nat. Rev. Neurol..

[111] Schapira A.H.V., Chaudhuri K.R., Jenner P. (2017). Non-motor features of Parkinson disease. Nat. Rev. Neurosci..

[112] Paredes-Rodriguez E., Vegas-Suarez S., Morera-Herreras T., De Deurwaerdere P., Miguelez C. (2020). The Noradrenergic System in Parkinson’s Disease. Front. Pharmacol..

[113] Kinnerup M.B., Sommerauer M., Damholdt M.F., Schaldemose J.L., Ismail R., Terkelsen A.J., Stær K., Hansen A., Fedorova T.D., Knudsen K. (2021). Preserved noradrenergic function in Parkinson’s disease patients with rest tremor. Neurobiol. Dis..

[114] Doppler C.E.J., Smit J.A.M., Hommelsen M., Seger A., Horsager J., Kinnerup M.B., Hansen A.K., Fedorova T.D., Knudsen K., Otto M. (2021). Microsleep disturbances are associated with noradrenergic dysfunction in Parkinson’s disease. Sleep.

[115] Helmich R.C., Lehéricy S. (2021). Dying-back of ascending noradrenergic projections in Parkinson’s disease. Brain.

[116] Müller M.L.T.M., Bohnen N.I. (2013). Cholinergic Dysfunction in Parkinson’s Disease. Curr. Neurol. Neurosci. Rep..

[117] Trujillo P., Song A.K., Hay K.R., Aumann M., Yan Y., Kang H., Donahue M.J., Claassen D.O. (2022). Dopamine-induced changes to thalamic GABA concentration in impulsive Parkinson disease patients. Npj Park. Dis..

[118] Terkelsen M.H., Hvingelby V.S., Pavese N. (2022). Molecular Imaging of the GABAergic System in Parkinson’s Disease and Atypical Parkinsonisms. Curr. Neurol. Neurosci. Rep..

[119] Alharbi B., Al-kuraishy H.M., Al-Gareeb A.I., Elekhnawy E., Alharbi H., Alexiou A., Papadakis M., Batiha G.E.-S. (2024). Role of GABA pathway in motor and non-motor symptoms in Parkinson’s disease: A bidirectional circuit. Eur. J. Med. Res..

[120] Schwarzschild M.A., Agnati L., Fuxe K., Chen J.-F., Morelli M. (2006). Targeting adenosine A2A receptors in Parkinson’s disease. Trends Neurosci..

[121] Hawkes C.H., Del Tredici K., Braak H. (2007). Parkinson’s disease: A dual-hit hypothesis. Neuropathol. Appl. Neurobiol..

[122] Wu Y., Le W., Jankovic J. (2011). Preclinical Biomarkers of Parkinson Disease. Arch. Neurol..

[123] Fazio P., Svenningsson P., Cselényi Z., Halldin C., Farde L., Varrone A. (2018). Nigrostriatal dopamine transporter availability in early Parkinson’s disease. Mov. Disord. Off. J. Mov. Disord. Soc..

[124] Liu S.-Y., Chan P., Stoessl A.J. (2017). The underlying mechanism of prodromal PD: Insights from the parasympathetic nervous system and the olfactory system. Transl. Neurodegener..

[125] Ahadiat S.-A., Hosseinian Z. (2023). A look back at the prodromal findings in Parkinson’s disease. Bull. Natl. Res. Cent..

[126] Landers M.R., Johnson K.N., Johnson S., Ormsby T., Salgo D.C., Zorn J.B., Lyle J., Murtishaw A.S., Salazar A.M., Kinney J.W. (2019). Pre-diagnosis physical activity habits are associated with age of diagnosis in Parkinson’s disease. Clin. Park. Relat. Disord..

[127] Landers M.R., Navalta J.W., Murtishaw A.S., Kinney J.W., Pirio Richardson S. (2019). A High-Intensity Exercise Boot Camp for Persons With Parkinson Disease: A Phase II, Pragmatic, Randomized Clinical Trial of Feasibility, Safety, Signal of Efficacy, and Disease Mechanisms. J. Neurol. Phys. Ther. JNPT.

[128] Picillo M., Moccia M., Spina E., Barone P., Pellecchia M.T. (2016). Biomarkers of Parkinson’s disease: Recent insights, current challenges, and future prospects. J. Park. Restless Legs Syndr..

[129] Fazlollahi A., Zahmatyar M., Alizadeh H., Noori M., Jafari N., Nejadghaderi S.A., Sullman M.J.M., Gharagozli K., Kolahi A.-A., Safiri S. (2022). Association between gout and the development of Parkinson’s disease: A systematic review and meta-analysis. BMC Neurol..

[130] Liu L., Han Y., Zhang Z., Wang Y., Hu Y., Kaznacheyeva E., Ding J., Guo D., Wang G., Li B. (2023). Loss of DJ-1 function contributes to Parkinson’s disease pathogenesis in mice via RACK1-mediated PKC activation and MAO-B upregulation. Acta Pharmacol. Sin..

[131] Sun W., Zheng J., Ma J., Wang Z., Shi X., Li M., Huang S., Hu S., Zhao Z., Li D. (2021). Increased Plasma Heme Oxygenase-1 Levels in Patients With Early-Stage Parkinson’s Disease. Front. Aging Neurosci..

[132] Song W., Kothari V., Velly A.M., Cressatti M., Liberman A., Gornitsky M., Schipper H.M. (2018). Evaluation of salivary heme oxygenase-1 as a potential biomarker of early Parkinson’s disease. Mov. Disord. Off. J. Mov. Disord. Soc..

[133] Galindez J.M., Juwara L., Cressatti M., Gornitsky M., Velly A.M., Schipper H.M. (2021). Salivary Heme Oxygenase-1: A Potential Biomarker for Central Neurodegeneration. J. Cent. Nerv. Syst. Dis..

[134] Du T., Wang L., Liu W., Zhu G., Chen Y., Zhang J. (2021). Biomarkers and the Role of α-Synuclein in Parkinson’s Disease. Front. Aging Neurosci..

[135] Yilmaz R., Hopfner F., van Eimeren T., Berg D. (2019). Biomarkers of Parkinson’s disease: 20 years later. J. Neural Transm. Vienna Austria 1996.

[136] Knudsen K., Krogh K., Østergaard K., Borghammer P. (2017). Constipation in parkinson’s disease: Subjective symptoms, objective markers, and new perspectives. Mov. Disord..

[137] Stirpe P., Hoffman M., Badiali D., Colosimo C. (2016). Constipation: An emerging risk factor for Parkinson’s disease?. Eur. J. Neurol..

[138] Simonet C., Bestwick J., Jitlal M., Waters S., Ben-Joseph A., Marshall C.R., Dobson R., Marrium S., Robson J., Jacobs B.M. (2022). Assessment of Risk Factors and Early Presentations of Parkinson Disease in Primary Care in a Diverse UK Population. JAMA Neurol..

[139] Patel B., Chiu S., Armstrong M.J. (2022). Identifying Parkinson Risk Markers in Primary Care-Old Associations and New Insights. JAMA Neurol..

[140] Miller-Patterson C., Hsu J.Y., Willis A.W., Hamedani A.G. (2023). Functional Impairment in Individuals With Prodromal or Unrecognized Parkinson Disease. JAMA Neurol..

[141] Mahlknecht P., Gasperi A., Willeit P., Kiechl S., Stockner H., Willeit J., Rungger G., Sawires M., Nocker M., Rastner V. (2016). Prodromal Parkinson’s disease as defined per MDS research criteria in the general elderly community. Mov. Disord. Off. J. Mov. Disord. Soc..

[142] Marini K., Mahlknecht P., Tutzer F., Stockner H., Gasperi A., Djamshidian A., Willeit P., Kiechl S., Willeit J., Rungger G. (2020). Application of a Simple Parkinson’s Disease Risk Score in a Longitudinal Population-Based Cohort. Mov. Disord. Off. J. Mov. Disord. Soc..

[143] Pilotto A., Heinzel S., Suenkel U., Lerche S., Brockmann K., Roeben B., Schaeffer E., Wurster I., Yilmaz R., Liepelt-Scarfone I. (2017). Application of the movement disorder society prodromal Parkinson’s disease research criteria in 2 independent prospective cohorts. Mov. Disord. Off. J. Mov. Disord. Soc..

[144] Plouvier A.O.A., Hameleers R.J.M.G., van den Heuvel E.A.J., Bor H.H., Olde Hartman T.C., Bloem B.R., van Weel C., Lagro-Janssen A.L.M. (2014). Prodromal symptoms and early detection of Parkinson’s disease in general practice: A nested case-control study. Fam. Pract..

[145] Heinzel S., Berg D., Gasser T., Chen H., Yao C., Postuma R.B. (2019). MDS Task Force on the Definition of Parkinson’s Disease Update of the MDS research criteria for prodromal Parkinson’s disease. Mov. Disord. Off. J. Mov. Disord. Soc..

[146] Noyce A.J., Bestwick J.P., Silveira-Moriyama L., Hawkes C.H., Knowles C.H., Hardy J., Giovannoni G., Nageshwaran S., Osborne C., Lees A.J. (2014). PREDICT-PD: Identifying risk of Parkinson’s disease in the community: Methods and baseline results. J. Neurol. Neurosurg. Psychiatry.

[147] Zhang J.D., Xue C., Kolachalama V.B., Donald W.A. (2023). Interpretable Machine Learning on Metabolomics Data Reveals Biomarkers for Parkinson’s Disease. ACS Cent. Sci..

[148] Berg D., Postuma R.B., Adler C.H., Bloem B.R., Chan P., Dubois B., Gasser T., Goetz C.G., Halliday G., Joseph L. (2015). MDS research criteria for prodromal Parkinson’s disease: MDS Criteria for Prodromal PD. Mov. Disord..

[149] Llido J.P., Jayanti S., Tiribelli C., Gazzin S. (2023). Bilirubin and Redox Stress in Age-Related Brain Diseases. Antioxidants.

[150] Macías-García D., Méndez-Del Barrio C., Jesús S., Labrador M.A., Adarmes-Gómez A., Vargas-González L., Carrillo F., Gómez-Garre P., Mir P. (2019). Increased bilirubin levels in Parkinson’s disease. Parkinsonism Relat. Disord..

[151] Moccia M., Picillo M., Erro R., Longo K., Amboni M., Santangelo G., Palladino R., Allocca R., Caporale O., Triassi M. (2015). Increased bilirubin levels in de novo Parkinson’s disease. Eur. J. Neurol..

[152] Albillos S.M., Montero O., Calvo S., Solano-Vila B., Trejo J.M., Cubo E. (2021). Plasma acyl-carnitines, bilirubin, tyramine and tetrahydro-21-deoxycortisol in Parkinson’s disease and essential tremor. A case control biomarker study. Parkinsonism Relat. Disord..

[153] Neely M.D., Schmidt D.E., Deutch A.Y. (2007). Cortical regulation of dopamine depletion-induced dendritic spine loss in striatal medium spiny neurons. Neuroscience.

[154] Dal Ben M., Bongiovanni R., Tuniz S., Fioriti E., Tiribelli C., Moretti R., Gazzin S. (2019). Earliest Mechanisms of Dopaminergic Neurons Sufferance in a Novel Slow Progressing Ex Vivo Model of Parkinson Disease in Rat Organotypic Cultures of Substantia Nigra. Int. J. Mol. Sci..

[155] Li Y., Yin Q., Wang B., Shen T., Luo W., Liu T. (2022). Preclinical reserpine models recapitulating motor and non-motor features of Parkinson’s disease: Roles of epigenetic upregulation of alpha-synuclein and autophagy impairment. Front. Pharmacol..

[156] Tieu K. (2011). A guide to neurotoxic animal models of Parkinson’s disease. Cold Spring Harb. Perspect. Med..

[157] Luchtman D.W., Shao D., Song C. (2009). Behavior, neurotransmitters and inflammation in three regimens of the MPTP mouse model of Parkinson’s disease. Physiol. Behav..

[158] Nayyar T., Bubser M., Ferguson M.C., Neely M.D., Goodwin J.S., Montine T.J., Deutch A.Y., Ansah T.A. (2009). Cortical serotonin and norepinephrine denervation in parkinsonism: Preferential loss of the beaded serotonin innervation. Eur. J. Neurosci..

[159] Ando R., Choudhury M.E., Yamanishi Y., Kyaw W.T., Kubo M., Kannou M., Nishikawa N., Tanaka J., Nomoto M., Nagai M. (2018). Modafinil alleviates levodopa-induced excessive nighttime sleepiness and restores monoaminergic systems in a nocturnal animal model of Parkinson’s disease. J. Pharmacol. Sci..

[160] Decourt M., Jiménez-Urbieta H., Benoit-Marand M., Fernagut P.-O. (2021). Neuropsychiatric and Cognitive Deficits in Parkinson’s Disease and Their Modeling in Rodents. Biomedicines.

[161] Lama J., Buhidma Y., Fletcher E.J.R., Duty S. (2021). Animal models of Parkinson’s disease: A guide to selecting the optimal model for your research. Neuronal Signal..

[162] Miyazaki I., Asanuma M. (2020). The Rotenone Models Reproducing Central and Peripheral Features of Parkinson’s Disease. NeuroSci.

[163] Taguchi T., Ikuno M., Yamakado H., Takahashi R. (2020). Animal Model for Prodromal Parkinson’s Disease. Int. J. Mol. Sci..

[164] Chaumette T., Lebouvier T., Aubert P., Lardeux B., Qin C., Li Q., Accary D., Bézard E., Bruley Des Varannes S., Derkinderen P. (2009). Neurochemical plasticity in the enteric nervous system of a primate animal model of experimental Parkinsonism. Neurogastroenterol. Motil..

[165] Drolet R.E., Cannon J.R., Montero L., Greenamyre J.T. (2009). Chronic rotenone exposure reproduces Parkinson’s disease gastrointestinal neuropathology. Neurobiol. Dis..

[166] Greene J.G., Noorian A.R., Srinivasan S. (2009). Delayed gastric emptying and enteric nervous system dysfunction in the rotenone model of Parkinson’s disease. Exp. Neurol..

[167] Real C.C., Binda K.H., Thomsen M.B., Lillethorup T.P., Brooks D.J., Landau A.M. (2023). Selecting the Best Animal Model of Parkinson’s Disease for Your Research Purpose: Insight from in vivo PET Imaging Studies. Curr. Neuropharmacol..

[168] Sulzer D. (2007). Multiple hit hypotheses for dopamine neuron loss in Parkinson’s disease. Trends Neurosci..

[169] Noyce A.J., Bestwick J.P., Silveira-Moriyama L., Hawkes C.H., Giovannoni G., Lees A.J., Schrag A. (2012). Meta-Analysis of Early Nonmotor Features and Risk Factors for Parkinson Disease. Ann. Neurol..

[170] Foubert-Samier A., Helmer C., Perez F., Le Goff M., Auriacombe S., Elbaz A., Dartigues J.-F., Tison F. (2012). Past exposure to neuroleptic drugs and risk of Parkinson disease in an elderly cohort. Neurology.

[171] Goldman S.M., Quinlan P.J., Ross G.W., Marras C., Meng C., Bhudhikanok G.S., Comyns K., Korell M., Chade A.R., Kasten M. (2012). Solvent Exposures and Parkinson’s Disease Risk in Twins. Ann. Neurol..

[172] Adams J.D. (2021). Possible causes of Parkinson’s disease. Front. Biosci. Landmark Ed..

[173] Chen H., Jacobs E., Schwarzschild M.A., McCullough M.L., Calle E.E., Thun M.J., Ascherio A. (2005). Nonsteroidal antiinflammatory drug use and the risk for Parkinson’s disease. Ann. Neurol..

[174] Martin-de-Pablos A., Córdoba-Fernández A., Fernández-Espejo E. (2018). Analysis of neurotrophic and antioxidant factors related to midbrain dopamine neuronal loss and brain inflammation in the cerebrospinal fluid of the elderly. Exp. Gerontol..

[175] Leite F., Ribeiro L. (2020). Dopaminergic Pathways in Obesity-Associated Inflammation. J. Neuroimmune Pharmacol..

[176] Doroszkiewicz J., Groblewska M., Mroczko B. (2021). The Role of sGut Microbiota and Gut-Brain Interplay in Selected Diseases of the Central Nervous System. Int. J. Mol. Sci..

[177] Kalampokini S., Becker A., Fassbender K., Lyros E., Unger M.M. (2019). Nonpharmacological Modulation of Chronic Inflammation in Parkinson’s Disease: Role of Diet Interventions. Park. Dis..

[178] Van Den Eeden S.K., Tanner C.M., Bernstein A.L., Fross R.D., Leimpeter A., Bloch D.A., Nelson L.M. (2003). Incidence of Parkinson’s disease: Variation by age, gender, and race/ethnicity. Am. J. Epidemiol..

[179] Nalls M.A., Pankratz N., Lill C.M., Do C.B., Hernandez D.G., Saad M., DeStefano A.L., Kara E., Bras J., Sharma M. (2014). Large-scale meta-analysis of genome-wide association data identifies six new risk loci for Parkinson’s disease. Nat. Genet..

[180] Trinh J., Farrer M. (2013). Advances in the genetics of Parkinson disease. Nat. Rev. Neurol..

[181] Magistrelli L., Contaldi E., Vignaroli F., Gallo S., Colombatto F., Cantello R., Comi C. (2022). Immune Response Modifications in the Genetic Forms of Parkinson’s Disease: What Do We Know?. Int. J. Mol. Sci..

[182] Furuyashiki T. (2012). Roles of dopamine and inflammation-related molecules in behavioral alterations caused by repeated stress. J. Pharmacol. Sci..

[183] Li M., Zhou L., Sun X., Yang Y., Zhang C., Wang T., Fu F. (2022). Dopamine, a co-regulatory component, bridges the central nervous system and the immune system. Biomed. Pharmacother..

[184] Marchetti B., Giachino C., Tirolo C., Serapide M.F. (2022). “Reframing” dopamine signaling at the intersection of glial networks in the aged Parkinsonian brain as innate Nrf2/Wnt driver: Therapeutical implications. Aging Cell.

[185] Feng Y., Lu Y. (2021). Immunomodulatory Effects of Dopamine in Inflammatory Diseases. Front. Immunol..

[186] Nolan Y.M., Sullivan A.M., Toulouse A. (2013). Parkinson’s disease in the nuclear age of neuroinflammation. Trends Mol. Med..

[187] Xia Q.-P., Cheng Z.-Y., He L. (2019). The modulatory role of dopamine receptors in brain neuroinflammation. Int. Immunopharmacol..

[188] Melis M., Carta G., Pistis M., Banni S. (2013). Physiological Role of Peroxisome Proliferator-Activated Receptors Type Alpha on Dopamine Systems. CNS Neurol. Disord.-Drug Targets-CNS Neurol. Disord..

[189] Pingale T., Gupta G.L. (2020). Classic and evolving animal models in Parkinson’s disease. Pharmacol. Biochem. Behav..

[190] Zeng X.-S., Geng W.-S., Jia J.-J. (2018). Neurotoxin-Induced Animal Models of Parkinson Disease: Pathogenic Mechanism and Assessment. ASN Neuro.

[191] Johnson M.E., Bobrovskaya L. (2015). An update on the rotenone models of Parkinson’s disease: Their ability to reproduce the features of clinical disease and model gene–environment interactions. NeuroToxicology.

[192] Innos J., Hickey M.A. (2021). Using Rotenone to Model Parkinson’s Disease in Mice: A Review of the Role of Pharmacokinetics. Chem. Res. Toxicol..

[193] Meredith G.E., Sonsalla P.K., Chesselet M.-F. (2008). Animal models of Parkinson’s disease progression. Acta Neuropathol..

[194] Deng I., Corrigan F., Zhai G., Zhou X.-F., Bobrovskaya L. (2020). Lipopolysaccharide animal models of Parkinson’s disease: Recent progress and relevance to clinical disease. Brain Behav. Immun.-Health.

[195] Klæstrup I.H., Just M.K., Holm K.L., Alstrup A.K.O., Romero-Ramos M., Borghammer P., Van Den Berge N. (2022). Impact of aging on animal models of Parkinson’s disease. Front. Aging Neurosci..

[196] Stahl S.M. (2018). Mechanism of action of vesicular monoamine transporter 2 (VMAT2) inhibitors in tardive dyskinesia: Reducing dopamine leads to less “go” and more “stop” from the motor striatum for robust therapeutic effects. CNS Spectr..

[197] Neha S., Ahmad M., Kumari B., Ali M.Z., Dholaniya P.S. (2022). Early Diagnosis of Parkinson’s Disease: Utility of Animal Models. Parkinson’s Disease—Animal Models, Current Therapies and Clinical Trials.

[198] Konnova E.A., Swanberg M., Stoker T.B., Greenland J.C. (2018). Animal Models of Parkinson’s Disease. Parkinson’s Disease: Pathogenesis and Clinical Aspects.

[199] Dovonou A., Bolduc C., Soto Linan V., Gora C., Peralta M.R., Lévesque M. (2023). Animal models of Parkinson’s disease: Bridging the gap between disease hallmarks and research questions. Transl. Neurodegener..

[200] de Freitas C.M., Busanello A., Schaffer L.F., Peroza L.R., Krum B.N., Leal C.Q., Ceretta A.P.C., da Rocha J.B.T., Fachinetto R. (2016). Behavioral and neurochemical effects induced by reserpine in mice. Psychopharmacology.

[201] Drukarch B., Jongenelen C.A., Schepens E., Langeveld C.H., Stoof J.C. (1996). Glutathione is involved in the granular storage of dopamine in rat PC 12 pheochromocytoma cells: Implications for the pathogenesis of Parkinson’s disease. J. Neurosci. Off. J. Soc. Neurosci..

[202] Goldstein D.S., Sullivan P., Cooney A., Jinsmaa Y., Sullivan R., Gross D.J., Holmes C., Kopin I.J., Sharabi Y. (2012). Vesicular uptake blockade generates the toxic dopamine metabolite 3,4-dihydroxyphenylacetaldehyde in PC12 cells: Relevance to the pathogenesis of Parkinson’s disease. J. Neurochem..

[203] Glinka Y., Gassen M., Youdim M.B. (1997). Mechanism of 6-hydroxydopamine neurotoxicity. J. Neural Transm. Suppl..

[204] Henning J., Strauss U., Wree A., Gimsa J., Rolfs A., Benecke R., Gimsa U. (2008). Differential astroglial activation in 6-hydroxydopamine models of Parkinson’s disease. Neurosci. Res..

[205] Blesa J., Przedborski S. (2014). Parkinson’s disease: Animal models and dopaminergic cell vulnerability. Front. Neuroanat..

[206] Hisahara S., Shimohama S. (2010). Toxin-Induced and Genetic Animal Models of Parkinson’s Disease. Park. Dis..

[207] Antunes M.S., Cattelan Souza L., Ladd F.V.L., Ladd A.A.B.L., Moreira A.L., Bortolotto V.C., Silva M.R.P., Araújo S.M., Prigol M., Nogueira C.W. (2020). Hesperidin Ameliorates Anxiety-Depressive-Like Behavior in 6-OHDA Model of Parkinson’s Disease by Regulating Striatal Cytokine and Neurotrophic Factors Levels and Dopaminergic Innervation Loss in the Striatum of Mice. Mol. Neurobiol..

[208] Khan E., Hasan I., Haque M.E. (2023). Parkinson’s Disease: Exploring Different Animal Model Systems. Int. J. Mol. Sci..

[209] Berger K., Przedborski S., Cadet J.L. (1991). Retrograde degeneration of nigrostriatal neurons induced by intrastriatal 6-hydroxydopamine injection in rats. Brain Res. Bull..

[210] Lou H., Jing X., Wei X., Shi H., Ren D., Zhang X. (2014). Naringenin protects against 6-OHDA-induced neurotoxicity via activation of the Nrf2/ARE signaling pathway. Neuropharmacology.

[211] Cousins M.S., Salamone J.D. (1996). Involvement of ventrolateral striatal dopamine in movement initiation and execution: A microdialysis and behavioral investigation. Neuroscience.

[212] Santiago R.M., Barbiero J., Gradowski R.W., Bochen S., Lima M.M.S., Da Cunha C., Andreatini R., Vital M.A.B.F. (2014). Induction of depressive-like behavior by intranigral 6-OHDA is directly correlated with deficits in striatal dopamine and hippocampal serotonin. Behav. Brain Res..

[213] Tadaiesky M.T., Dombrowski P.A., Figueiredo C.P., Cargnin-Ferreira E., Da Cunha C., Takahashi R.N. (2008). Emotional, cognitive and neurochemical alterations in a premotor stage model of Parkinson’s disease. Neuroscience.

[214] Anichtchik O.V., Kaslin J., Peitsaro N., Scheinin M., Panula P. (2004). Neurochemical and behavioural changes in zebrafish Danio rerio after systemic administration of 6-hydroxydopamine and 1-methyl-4-phenyl-1,2,3,6-tetrahydropyridine. J. Neurochem..

[215] Lindner M.D., Plone M.A., Francis J.M., Blaney T.J., Salamone J.D., Emerich D.F. (1997). Rats with partial striatal dopamine depletions exhibit robust and long-lasting behavioral deficits in a simple fixed-ratio bar-pressing task. Behav. Brain Res..

[216] Kamińska K., Lenda T., Konieczny J., Czarnecka A., Lorenc-Koci E. (2017). Depressive-like neurochemical and behavioral markers of Parkinson’s disease after 6-OHDA administered unilaterally to the rat medial forebrain bundle. Pharmacol. Rep..

[217] Feng C.-W., Wen Z.-H., Huang S.-Y., Hung H.-C., Chen C.-H., Yang S.-N., Chen N.-F., Wang H.-M., Hsiao C.-D., Chen W.-F. (2014). Effects of 6-hydroxydopamine exposure on motor activity and biochemical expression in zebrafish (Danio rerio) larvae. Zebrafish.

[218] Loiodice S., Wing Young H., Rion B., Méot B., Montagne P., Denibaud A.-S., Viel R., Drieu La Rochelle C. (2019). Implication of nigral dopaminergic lesion and repeated L-dopa exposure in neuropsychiatric symptoms of Parkinson’s disease. Behav. Brain Res..

[219] Na S.J., DiLella A.G., Lis E.V., Jones K., Levine D.M., Stone D.J., Hess J.F. (2010). Molecular profiling of a 6-hydroxydopamine model of Parkinson’s disease. Neurochem. Res..

[220] Walsh S., Finn D.P., Dowd E. (2011). Time-course of nigrostriatal neurodegeneration and neuroinflammation in the 6-hydroxydopamine-induced axonal and terminal lesion models of Parkinson’s disease in the rat. Neuroscience.

[221] Parng C., Roy N.M., Ton C., Lin Y., McGrath P. (2007). Neurotoxicity assessment using zebrafish. J. Pharmacol. Toxicol. Methods.

[222] Sauer H., Oertel W.H. (1994). Progressive degeneration of nigrostriatal dopamine neurons following intrastriatal terminal lesions with 6-hydroxydopamine: A combined retrograde tracing and immunocytochemical study in the rat. Neuroscience.

[223] Alzoubi K.H., Mokhemer E., Abuirmeileh A.N. (2018). Beneficial effect of etazolate on depression-like behavior and, learning, and memory impairment in a model of Parkinson’s disease. Behav. Brain Res..

[224] Vo Q., Gilmour T.P., Venkiteswaran K., Fang J., Subramanian T. (2014). Polysomnographic Features of Sleep Disturbances and REM Sleep Behavior Disorder in the Unilateral 6-OHDA Lesioned Hemiparkinsonian Rat. Park. Dis..

[225] Jordán J., Galindo M.F., Tornero D., González-García C., Ceña V. (2004). Bcl-xL blocks mitochondrial multiple conductance channel activation and inhibits 6-OHDA-induced death in SH-SY5Y cells. J. Neurochem..

[226] Choi W.-S., Eom D.-S., Han B.S., Kim W.K., Han B.H., Choi E.-J., Oh T.H., Markelonis G.J., Cho J.W., Oh Y.J. (2004). Phosphorylation of p38 MAPK Induced by Oxidative Stress Is Linked to Activation of Both Caspase-8- and -9-mediated Apoptotic Pathways in Dopaminergic Neurons. J. Biol. Chem..

[227] Pieńkowska N., Bartosz G., Sadowska-Bartosz I. (2023). Effect of 6-hydroxydopamine increase the glutathione level in SH-SY5Y human neuroblastoma cells. Acta Biochim. Pol..

[228] Ouyang M., Shen X. (2006). Critical role of ASK1 in the 6-hydroxydopamine-induced apoptosis in human neuroblastoma SH-SY5Y cells. J. Neurochem..

[229] Huang Z., Han J., Wu P., Wu C., Fan Y., Zhao L., Hao X., Chen D., Zhu M. (2022). Sorting Nexin 5 Plays an Important Role in Promoting Ferroptosis in Parkinson’s Disease. Oxid. Med. Cell. Longev..

[230] Tian Y., Lu J., Hao X., Li H., Zhang G., Liu X., Li X., Zhao C., Kuang W., Chen D. (2020). FTH1 Inhibits Ferroptosis Through Ferritinophagy in the 6-OHDA Model of Parkinson’s Disease. Neurotherapeutics.

[231] Lee Y.M., Park S.H., Shin D.-I., Hwang J.-Y., Park B., Park Y.-J., Lee T.H., Chae H.Z., Jin B.K., Oh T.H. (2008). Oxidative Modification of Peroxiredoxin Is Associated with Drug-induced Apoptotic Signaling in Experimental Models of Parkinson Disease. J. Biol. Chem..

[232] Oh C.-K., Choi Y.K., Hwang I.-Y., Ko Y.U., Chung I.K., Yun N., Oh Y.J. (2020). RING-finger protein 166 plays a novel pro-apoptotic role in neurotoxin-induced neurodegeneration via ubiquitination of XIAP. Cell Death Dis..

[233] Han B.S., Hong H.-S., Choi W.-S., Markelonis G.J., Oh T.H., Oh Y.J. (2003). Caspase-Dependent and -Independent Cell Death Pathways in Primary Cultures of Mesencephalic Dopaminergic Neurons after Neurotoxin Treatment. J. Neurosci..

[234] Jang H.J., Chung K.C. (2022). The ubiquitin-proteasome system and autophagy mutually interact in neurotoxin-induced dopaminergic cell death models of Parkinson’s disease. FEBS Lett..

[235] Lei C., Zhongyan Z., Wenting S., Jing Z., Liyun Q., Hongyi H., Juntao Y., Qing Y. (2023). Identification of necroptosis-related genes in Parkinson’s disease by integrated bioinformatics analysis and experimental validation. Front. Neurosci..

[236] Gerlach M., Riederer P., Przuntek H., Youdim M.B. (1991). MPTP mechanisms of neurotoxicity and their implications for Parkinson’s disease. Eur. J. Pharmacol..

[237] Jackson-Lewis V., Blesa J., Przedborski S. (2012). Animal models of Parkinson’s disease. Parkinsonism Relat. Disord..

[238] Yu Q., Huang Q., Du X., Xu S., Li M., Ma S. (2018). Early activation of Egr-1 promotes neuroinflammation and dopaminergic neurodegeneration in an experimental model of Parkinson’s disease. Exp. Neurol..

[239] Lofrumento D.D., Saponaro C., Cianciulli A., De Nuccio F., Mitolo V., Nicolardi G., Panaro M.A. (2011). MPTP-induced neuroinflammation increases the expression of pro-inflammatory cytokines and their receptors in mouse brain. Neuroimmunomodulation.

[240] Giovanni A., Sieber B.A., Heikkila R.E., Sonsalla P.K. (1994). Studies on species sensitivity to the dopaminergic neurotoxin 1-methyl-4-phenyl-1,2,3,6-tetrahydropyridine. Part 1: Systemic administration. J. Pharmacol. Exp. Ther..

[241] Han N.-R., Kim Y.-K., Ahn S., Hwang T.-Y., Lee H., Park H.-J. (2020). A Comprehensive Phenotype of Non-motor Impairments and Distribution of Alpha-Synuclein Deposition in Parkinsonism-Induced Mice by a Combination Injection of MPTP and Probenecid. Front. Aging Neurosci..

[242] Cunha M.P., Pazini F.L., Lieberknecht V., Budni J., Oliveira Á., Rosa J.M., Mancini G., Mazzardo L., Colla A.R., Leite M.C. (2017). MPP+-Lesioned Mice: An Experimental Model of Motor, Emotional, Memory/Learning, and Striatal Neurochemical Dysfunctions. Mol. Neurobiol..

[243] Shi J., Cai Q., Zhang J., He X., Liu Y., Zhu R., Jin L. (2017). AM1241 alleviates MPTP-induced Parkinson’s disease and promotes the regeneration of DA neurons in PD mice. Oncotarget.

[244] Lu Y., Zhang X., Zhao L., Yang C., Pan L., Li C., Liu K., Bai G., Gao H., Yan Z. (2018). Metabolic Disturbances in the Striatum and Substantia Nigra in the Onset and Progression of MPTP-Induced Parkinsonism Model. Front. Neurosci..

[245] Omar N.A., Kumar J., Teoh S.L. (2023). Parkinson’s disease model in zebrafish using intraperitoneal MPTP injection. Front. Neurosci..

[246] Bezard E., Imbert C., Deloire X., Bioulac B., Gross C.E. (1997). A chronic MPTP model reproducing the slow evolution of Parkinson’s disease: Evolution of motor symptoms in the monkey. Brain Res..

[247] Pifl C., Schingnitz G., Hornykiewicz O. (1991). Effect of 1-methyl-4-phenyl-1,2,3,6-tetrahydropyridine on the regional distribution of brain monoamines in the rhesus monkey. Neuroscience.

[248] Graham W.C., Robertson R.G., Sambrook M.A., Crossman A.R. (1990). Injection of excitatory amino acid antagonists into the medial pallidal segment of a 1-methyl-4-phenyl-1,2,3,6-tetrahydropyridine (MPTP) treated primate reverses motor symptoms of parkinsonism. Life Sci..

[249] Iravani M.M., Syed E., Jackson M.J., Johnston L.C., Smith L.A., Jenner P. (2005). A modified MPTP treatment regime produces reproducible partial nigrostriatal lesions in common marmosets. Eur. J. Neurosci..

[250] Vucković M.G., Wood R.I., Holschneider D.P., Abernathy A., Togasaki D.M., Smith A., Petzinger G.M., Jakowec M.W. (2008). Memory, mood, dopamine, and serotonin in the 1-methyl-4-phenyl-1,2,3,6-tetrahydropyridine-lesioned mouse model of basal ganglia injury. Neurobiol. Dis..

[251] Wen L., Wei W., Gu W., Huang P., Ren X., Zhang Z., Zhu Z., Lin S., Zhang B. (2008). Visualization of monoaminergic neurons and neurotoxicity of MPTP in live transgenic zebrafish. Dev. Biol..

[252] Lam C.S., Korzh V., Strahle U. (2005). Zebrafish embryos are susceptible to the dopaminergic neurotoxin MPTP. Eur. J. Neurosci..

[253] Sarath Babu N., Murthy C.L.N., Kakara S., Sharma R., Brahmendra Swamy C.V., Idris M.M. (2016). 1-Methyl-4-phenyl-1,2,3,6-tetrahydropyridine induced Parkinson’s disease in zebrafish. Proteomics.

[254] Tang L., Xu N., Huang M., Yi W., Sang X., Shao M., Li Y., Hao Z., Liu R., Shen Y. (2023). A primate nigrostriatal atlas of neuronal vulnerability and resilience in a model of Parkinson’s disease. Nat. Commun..

[255] Ferro M.M., Bellissimo M.I., Anselmo-Franci J.A., Angellucci M.E.M., Canteras N.S., Da Cunha C. (2005). Comparison of bilaterally 6-OHDA- and MPTP-lesioned rats as models of the early phase of Parkinson’s disease: Histological, neurochemical, motor and memory alterations. J. Neurosci. Methods.

[256] Schneider J.S., Roeltgen D.P. (1993). Delayed matching-to-sample, object retrieval, and discrimination reversal deficits in chronic low dose MPTP-treated monkeys. Brain Res..

[257] Mandel S., Grünblatt E., Maor G., Youdim M.B.H. (2002). Early and Late Gene Changes in MPTP Mice Model of Parkinson’s Disease Employing cDNA Microarray. Neurochem. Res..

[258] Benner E.J., Mosley R.L., Destache C.J., Lewis T.B., Jackson-Lewis V., Gorantla S., Nemachek C., Green S.R., Przedborski S., Gendelman H.E. (2004). Therapeutic immunization protects dopaminergic neurons in a mouse model of Parkinson’s disease. Proc. Natl. Acad. Sci. USA.

[259] Barraud Q., Lambrecq V., Forni C., McGuire S., Hill M., Bioulac B., Balzamo E., Bezard E., Tison F., Ghorayeb I. (2009). Sleep disorders in Parkinson’s disease: The contribution of the MPTP non-human primate model. Exp. Neurol..

[260] Verhave P.S., Jongsma M.J., Van den Berg R.M., Vis J.C., Vanwersch R.A.P., Smit A.B., Van Someren E.J.W., Philippens I.H.C.H.M. (2011). REM Sleep Behavior Disorder in the Marmoset MPTP Model of Early Parkinson Disease. Sleep.

[261] Forno L.S., Langston J.W., DeLanney L.E., Irwin I., Ricaurte G.A. (1986). Locus ceruleus lesions and eosinophilic inclusions in MPTP-treated monkeys. Ann. Neurol..

[262] Forno L.S., DeLanney L.E., Irwin I., Langston J.W. (1993). Similarities and differences between MPTP-induced parkinsonsim and Parkinson’s disease. Neuropathologic considerations. Adv. Neurol..

[263] Martins J.B., Bastos M.D.L., Carvalho F., Capela J.P. (2013). Differential Effects of Methyl-4-Phenylpyridinium Ion, Rotenone, and Paraquat on Differentiated SH-SY5Y Cells. J. Toxicol..

[264] Dai H.-Y., Chang M.-X., Sun L. (2023). HOTAIRM1 knockdown reduces MPP+-induced oxidative stress injury of SH-SY5Y cells by activating the Nrf2/HO-1 pathway. Transl. Neurosci..

[265] Krug A.K., Gutbier S., Zhao L., Pöltl D., Kullmann C., Ivanova V., Förster S., Jagtap S., Meiser J., Leparc G. (2014). Transcriptional and metabolic adaptation of human neurons to the mitochondrial toxicant MPP+. Cell Death Dis..

[266] Shindo Y., Yamanaka R., Suzuki K., Hotta K., Oka K. (2016). Altered expression of Mg^2+^ transport proteins during Parkinson’s disease-like dopaminergic cell degeneration in PC12 cells. Biochim. Biophys. Acta.

[267] Xiao X., Tan Z., Jia M., Zhou X., Wu K., Ding Y., Li W. (2021). Long Noncoding RNA SNHG1 Knockdown Ameliorates Apoptosis, Oxidative Stress and Inflammation in Models of Parkinson&rsquo;s Disease by Inhibiting the miR-125b-5p/MAPK1 Axis. Neuropsychiatr. Dis. Treat..

[268] He S., Wang Q., Chen L., He Y.J., Wang X., Qu S. (2023). miR-100a-5p-enriched exosomes derived from mesenchymal stem cells enhance the anti-oxidant effect in a Parkinson’s disease model via regulation of Nox4/ROS/Nrf2 signaling. J. Transl. Med..

[269] Youdim M.B.H., Grünblatt E., Levites Y., Maor G., Mandel S. (2002). Early and late molecular events in neurodegeneration and neuroprotection in Parkinson’s disease MPTP model as assessed by cDNA microarray; the role of iron. Neurotox. Res..

[270] Liu T., Wang P., Yin H., Wang X., Lv J., Yuan J., Zhu J., Wang Y. (2023). Rapamycin reverses ferroptosis by increasing autophagy in MPTP/MPP+-induced models of Parkinson’s disease. Neural Regen. Res..

[271] Ren J., Zhao Y., Sun X. (2009). Toxic influence of chronic oral administration of paraquat on nigrostriatal dopaminergic neurons in C57BL/6 mice. Chin. Med. J..

[272] Bortolotto J.W., Cognato G.P., Christoff R.R., Roesler L.N., Leite C.E., Kist L.W., Bogo M.R., Vianna M.R., Bonan C.D. (2014). Long-term exposure to paraquat alters behavioral parameters and dopamine levels in adult zebrafish (*Danio rerio*). Zebrafish.

[273] Muthukumaran K., Leahy S., Harrison K., Sikorska M., Sandhu J.K., Cohen J., Keshan C., Lopatin D., Miller H., Borowy-Borowski H. (2014). Orally delivered water soluble Coenzyme Q10 (Ubisol-Q10) blocks on-going neurodegeneration in rats exposed to paraquat: Potential for therapeutic application in Parkinson’s disease. BMC Neurosci..

[274] Nellore J., Nandita P. (2015). Paraquat exposure induces behavioral deficits in larval zebrafish during the window of dopamine neurogenesis. Toxicol. Rep..

[275] Vornov J.J., Park J., Thomas A.G. (1998). Regional vulnerability to endogenous and exogenous oxidative stress in organotypic hippocampal culture. Exp. Neurol..

[276] Wang F., Franco R., Skotak M., Hu G., Chandra N. (2014). Mechanical stretch exacerbates the cell death in SH-SY5Y cells exposed to paraquat: Mitochondrial dysfunction and oxidative stress. NeuroToxicology.

[277] Zuo Y., Xie J., Li X., Li Y., Thirupathi A., Zhang J., Yu P., Gao G., Chang Y., Shi Z. (2021). Ferritinophagy-Mediated Ferroptosis Involved in Paraquat-Induced Neurotoxicity of Dopaminergic Neurons: Implication for Neurotoxicity in PD. Oxid. Med. Cell. Longev..

[278] Ascherio A., Chen H., Weisskopf M.G., O’Reilly E., McCullough M.L., Calle E.E., Schwarzschild M.A., Thun M.J. (2006). Pesticide exposure and risk for Parkinson’s disease. Ann. Neurol..

[279] Dick F.D., De Palma G., Ahmadi A., Scott N.W., Prescott G.J., Bennett J., Semple S., Dick S., Counsell C., Mozzoni P. (2007). Environmental risk factors for Parkinson’s disease and parkinsonism: The Geoparkinson study. Occup. Environ. Med..

[280] Kang H., Han B.-S., Kim S.-J., Oh Y.J. (2012). Mechanisms to prevent caspase activation in rotenone-induced dopaminergic neurodegeneration: Role of ATP depletion and procaspase-9 degradation. Apoptosis.

[281] Yuyun X., Jinjun Q., Minfang X., Jing Q., Juan X., Rui M., Li Z., Jing G. (2013). Effects of Low Concentrations of Rotenone upon Mitohormesis in SH-SY5Y Cells. Dose-Response Publ. Int. Hormesis Soc..

[282] Van Laar A.D., Webb K.R., Keeney M.T., Van Laar V.S., Zharikov A., Burton E.A., Hastings T.G., Glajch K.E., Hirst W.D., Greenamyre J.T. (2023). Transient exposure to rotenone causes degeneration and progressive parkinsonian motor deficits, neuroinflammation, and synucleinopathy. Npj Park. Dis..

[283] Xu L., Hao L.-P., Yu J., Cheng S.-Y., Li F., Ding S.-M., Zhang R. (2023). Curcumin protects against rotenone-induced Parkinson’s disease in mice by inhibiting microglial NLRP3 inflammasome activation and alleviating mitochondrial dysfunction. Heliyon.

[284] Sanfeliu C., Bartra C., Suñol C., Rodríguez-Farré E. (2023). New insights in animal models of neurotoxicity-induced neurodegeneration. Front. Neurosci..

[285] Cannon J.R., Tapias V., Na H.M., Honick A.S., Drolet R.E., Greenamyre J.T. (2009). A highly reproducible rotenone model of Parkinson’s disease. Neurobiol. Dis..

[286] Chen Y., Zhang D., Liao Z., Wang B., Gong S., Wang C., Zhang M., Wang G., Cai H., Liao F.-F. (2015). Anti-oxidant polydatin (piceid) protects against substantia nigral motor degeneration in multiple rodent models of Parkinson’s disease. Mol. Neurodegener..

[287] Heikkila R.E., Nicklas W.J., Vyas I., Duvoisin R.C. (1985). Dopaminergic toxicity of rotenone and the 1-methyl-4-phenylpyridinium ion after their stereotaxic administration to rats: Implication for the mechanism of 1-methyl-4-phenyl-1,2,3,6-tetrahydropyridine toxicity. Neurosci. Lett..

[288] Rocha S.M., Bantle C.M., Aboellail T., Chatterjee D., Smeyne R.J., Tjalkens R.B. (2022). Rotenone induces regionally distinct α-synuclein protein aggregation and activation of glia prior to loss of dopaminergic neurons in C57Bl/6 mice. Neurobiol. Dis..

[289] Broome S.T., Musumeci G., Castorina A. (2022). PACAP and VIP Mitigate Rotenone-Induced Inflammation in BV-2 Microglial Cells. J. Mol. Neurosci..

[290] Sarkar S., Malovic E., Harishchandra D.S., Ghaisas S., Panicker N., Charli A., Palanisamy B.N., Rokad D., Jin H., Anantharam V. (2017). Mitochondrial impairment in microglia amplifies NLRP3 inflammasome proinflammatory signaling in cell culture and animal models of Parkinson’s disease. Npj Park. Dis..

[291] Zhang H., Yang J., Guo Y., Lü P., Gong X., Chen K., Li X., Tang M. (2024). Rotenone-induced PINK1/Parkin-mediated mitophagy: Establishing a silkworm model for Parkinson’s disease potential. Front. Mol. Neurosci..

[292] Wang L., Liu L., Han C., Jiang H., Ma K., Guo S., Xia Y., Wan F., Huang J., Xiong N. (2023). Histone Deacetylase 4 Inhibition Reduces Rotenone-Induced Alpha-Synuclein Accumulation via Autophagy in SH-SY5Y Cells. Brain Sci..

[293] Zhang L.-M., Wang M.-H., Yang H.-C., Tian T., Sun G.-F., Ji Y.-F., Hu W.-T., Liu X., Wang J.-P., Lu H. (2019). Dopaminergic neuron injury in Parkinson’s disease is mitigated by interfering lncRNA SNHG14 expression to regulate the miR-133b/α-synuclein pathway. Aging.

[294] Geng X., Zou Y., Li S., Qi R., Yu H., Li J. (2023). MALAT1 Mediates α-Synuclein Expression through miR-23b-3p to Induce Autophagic Impairment and the Inflammatory Response in Microglia to Promote Apoptosis in Dopaminergic Neuronal Cells. Mediators Inflamm..

[295] Liu Q., Li Q., Zhang R., Wang H., Li Y., Liu Z., Xie W., Geng D., Wang L. (2022). circ-Pank1 promotes dopaminergic neuron neurodegeneration through modulating miR-7a-5p/α-syn pathway in Parkinson’s disease. Cell Death Dis..

[296] Goldstein D.S., Sullivan P., Cooney A., Jinsmaa Y., Kopin I.J., Sharabi Y. (2015). Rotenone decreases intracellular aldehyde dehydrogenase activity: Implications for the pathogenesis of Parkinson’s disease. J. Neurochem..

[297] Jayanti S., Moretti R., Tiribelli C., Gazzin S. (2022). Bilirubin Prevents the TH+ Dopaminergic Neuron Loss in a Parkinson’s Disease Model by Acting on TNF-α. Int. J. Mol. Sci..

[298] Wang J., Wu W.-Y., Huang H., Li W.-Z., Chen H.-Q., Yin Y.-Y. (2016). Biochanin A Protects Against Lipopolysaccharide-Induced Damage of Dopaminergic Neurons Both In Vivo and In Vitro via Inhibition of Microglial Activation. Neurotox. Res..

[299] Fu S.-P., Wang J.-F., Xue W.-J., Liu H.-M., Liu B., Zeng Y.-L., Li S.-N., Huang B.-X., Lv Q.-K., Wang W. (2015). Anti-inflammatory effects of BHBA in both in vivo and in vitro Parkinson’s disease models are mediated by GPR109A-dependent mechanisms. J. Neuroinflamm..

[300] Hritcu L., Gorgan L.D. (2014). Intranigral lipopolysaccharide induced anxiety and depression by altered BDNF mRNA expression in rat hippocampus. Prog. Neuropsychopharmacol. Biol. Psychiatry.

[301] Hritcu L., Ciobica A. (2013). Intranigral lipopolysaccharide administration induced behavioral deficits and oxidative stress damage in laboratory rats: Relevance for Parkinson’s disease. Behav. Brain Res..

[302] He Q., Yu W., Wu J., Chen C., Lou Z., Zhang Q., Zhao J., Wang J., Xiao B. (2013). Intranasal LPS-mediated Parkinson’s model challenges the pathogenesis of nasal cavity and environmental toxins. PLoS ONE.

[303] Chen G., Liu J., Jiang L., Ran X., He D., Li Y., Huang B., Wang W., Fu S. (2018). Galangin Reduces the Loss of Dopaminergic Neurons in an LPS-Evoked Model of Parkinson’s Disease in Rats. Int. J. Mol. Sci..

[304] Qin L., Wu X., Block M.L., Liu Y., Breese G.R., Hong J.-S., Knapp D.J., Crews F.T. (2007). Systemic LPS causes chronic neuroinflammation and progressive neurodegeneration. Glia.

[305] Choi D.-Y., Liu M., Hunter R.L., Cass W.A., Pandya J.D., Sullivan P.G., Shin E.-J., Kim H.-C., Gash D.M., Bing G. (2009). Striatal neuroinflammation promotes Parkinsonism in rats. PLoS ONE.

[306] Ling Z., Gayle D.A., Ma S.Y., Lipton J.W., Tong C.W., Hong J.-S., Carvey P.M. (2002). In utero bacterial endotoxin exposure causes loss of tyrosine hydroxylase neurons in the postnatal rat midbrain. Mov. Disord. Off. J. Mov. Disord. Soc..

[307] Zheng L.-F., Zhang Y., Chen C.-L., Song J., Fan R.-F., Cai Q.-Q., Wang Z.-Y., Zhu J.-X. (2013). Alterations in TH- and ChAT-immunoreactive neurons in the DMV and gastric dysmotility in an LPS-induced PD rat model. Auton. Neurosci. Basic Clin..

[308] Qin L., Liu Y., Hong J.-S., Crews F.T. (2013). NADPH oxidase and aging drive microglial activation, oxidative stress and dopaminergic neurodegeneration following systemic LPS administration. Glia.

[309] Zheng H.-F., Yang Y.-P., Hu L.-F., Wang M.-X., Wang F., Cao L.-D., Li D., Mao C.-J., Xiong K.-P., Wang J.-D. (2013). Autophagic impairment contributes to systemic inflammation-induced dopaminergic neuron loss in the midbrain. PLoS ONE.

[310] Lai T.T., Kim Y.J., Nguyen P.T., Koh Y.H., Nguyen T.T., Ma H.-I., Kim Y.E. (2021). Temporal Evolution of Inflammation and Neurodegeneration With Alpha-Synuclein Propagation in Parkinson’s Disease Mouse Model. Front. Integr. Neurosci..

[311] Oliveras-Salvá M., Van der Perren A., Casadei N., Stroobants S., Nuber S., D’Hooge R., Van den Haute C., Baekelandt V. (2013). rAAV2/7 vector-mediated overexpression of alpha-synuclein in mouse substantia nigra induces protein aggregation and progressive dose-dependent neurodegeneration. Mol. Neurodegener..

[312] Sznejder-Pachołek A., Joniec-Maciejak I., Wawer A., Ciesielska A., Mirowska-Guzel D. (2017). The effect of α-synuclein on gliosis and IL-1α, TNFα, IFNγ, TGFβ expression in murine brain. Pharmacol. Rep..

[313] Jagmag S.A., Tripathi N., Shukla S.D., Maiti S., Khurana S. (2016). Evaluation of Models of Parkinson’s Disease. Front. Neurosci..

[314] Breger L.S., Fuzzati Armentero M.T. (2019). Genetically engineered animal models of Parkinson’s disease: From worm to rodent. Eur. J. Neurosci..

[315] Fathi M., Vakili K., Yaghoobpoor S., Qadirifard M.S., Kosari M., Naghsh N., Asgari taei A., Klegeris A., Dehghani M., Bahrami A. (2022). Pre-clinical Studies Identifying Molecular Pathways of Neuroinflammation in Parkinson’s Disease: A Systematic Review. Front. Aging Neurosci..

[316] Devine M.J., Ryten M., Vodicka P., Thomson A.J., Burdon T., Houlden H., Cavaleri F., Nagano M., Drummond N.J., Taanman J.-W. (2011). Parkinson’s disease induced pluripotent stem cells with triplication of the α-synuclein locus. Nat. Commun..

[317] Nguyen H.N., Byers B., Cord B., Shcheglovitov A., Byrne J., Gujar P., Kee K., Schüle B., Dolmetsch R.E., Langston W. (2011). LRRK2 mutant iPSC-derived DA neurons demonstrate increased susceptibility to oxidative stress. Cell Stem Cell.

[318] Razali K., Othman N., Mohd Nasir M.H., Doolaanea A.A., Kumar J., Ibrahim W.N., Mohamed Ibrahim N., Mohamed W.M.Y. (2021). The Promise of the Zebrafish Model for Parkinson’s Disease: Today’s Science and Tomorrow’s Treatment. Front. Genet..

[319] Vaz R.L., Outeiro T.F., Ferreira J.J. (2018). Zebrafish as an Animal Model for Drug Discovery in Parkinson’s Disease and Other Movement Disorders: A Systematic Review. Front. Neurol..

[320] Hughes G.L., Lones M.A., Bedder M., Currie P.D., Smith S.L., Pownall M.E. (2020). Machine learning discriminates a movement disorder in a zebrafish model of Parkinson’s disease. Dis. Model. Mech..

[321] Wasel O., Freeman J.L. (2020). Chemical and Genetic Zebrafish Models to Define Mechanisms of and Treatments for Dopaminergic Neurodegeneration. Int. J. Mol. Sci..

[322] Makhija D.T., Jagtap A.G. (2014). Studies on sensitivity of zebrafish as a model organism for Parkinson’s disease: Comparison with rat model. J. Pharmacol. Pharmacother..

[323] Thomas Broome S., Castorina A. (2022). Systemic Rotenone Administration Causes Extra-Nigral Alterations in C57BL/6 Mice. Biomedicines.

[324] Wang Q., Liu Y., Zhou J. (2015). Neuroinflammation in Parkinson’s disease and its potential as therapeutic target. Transl. Neurodegener..

[325] Lv D.-J., Li L.-X., Chen J., Wei S.-Z., Wang F., Hu H., Xie A.-M., Liu C.-F. (2019). Sleep deprivation caused a memory defects and emotional changes in a rotenone-based zebrafish model of Parkinson’s disease. Behav. Brain Res..

[326] Ramli M.D.B.C., Hashim N.H.B., Uzid M.B.M., Weinheimeri A.Z. (2020). Zebrafish parkinson’s model: The effects of tocotrienol rich fraction towards rotenone induced zebrafish. Int. J. Med. Toxicol. Leg. Med..

[327] Ding F., Luan L., Ai Y., Walton A., Gerhardt G.A., Gash D.M., Grondin R., Zhang Z. (2008). Development of a stable, early stage unilateral model of Parkinson’s disease in middle-aged rhesus monkeys. Exp. Neurol..

[328] Atack J.R., Shook B.C., Rassnick S., Jackson P.F., Rhodes K., Drinkenburg W.H., Ahnaou A., Te Riele P., Langlois X., Hrupka B. (2014). JNJ-40255293, a novel adenosine A2A/A1 antagonist with efficacy in preclinical models of Parkinson’s disease. ACS Chem. Neurosci..

[329] Ryu E.J., Harding H.P., Angelastro J.M., Vitolo O.V., Ron D., Greene L.A. (2002). Endoplasmic reticulum stress and the unfolded protein response in cellular models of Parkinson’s disease. J. Neurosci. Off. J. Soc. Neurosci..

[330] Xie H., Hu H., Chang M., Huang D., Gu X., Xiong X., Xiong R., Hu L., Li G. (2016). Identification of chaperones in a MPP+-induced and ATRA/TPA-differentiated SH-SY5Y cell PD model. Am. J. Transl. Res..

[331] Cenci M.A., Björklund A. (2020). Animal models for preclinical Parkinson’s research: An update and critical appraisal. Prog. Brain Res..

[332] Nair V.D., McNaught K.S.P., González-Maeso J., Sealfon S.C., Olanow C.W. (2006). p53 mediates nontranscriptional cell death in dopaminergic cells in response to proteasome inhibition. J. Biol. Chem..

[333] Lopes U.G., Erhardt P., Yao R., Cooper G.M. (1997). p53-dependent induction of apoptosis by proteasome inhibitors. J. Biol. Chem..

[334] Perez-Alvarez S., Solesio M.E., Manzanares J., Jordán J., Galindo M.F. (2009). Lactacystin requires reactive oxygen species and Bax redistribution to induce mitochondria-mediated cell death. Br. J. Pharmacol..

[335] Lee C.S., Han E.S., Park E.S., Bang H. (2005). Inhibition of MG132-induced mitochondrial dysfunction and cell death in PC12 cells by 3-morpholinosydnonimine. Brain Res..

[336] Bir A., Sen O., Anand S., Khemka V.K., Banerjee P., Cappai R., Sahoo A., Chakrabarti S. (2014). α-synuclein-induced mitochondrial dysfunction in isolated preparation and intact cells: Implications in the pathogenesis of Parkinson’s disease. J. Neurochem..

[337] Yamamoto N., Sawada H., Izumi Y., Kume T., Katsuki H., Shimohama S., Akaike A. (2007). Proteasome inhibition induces glutathione synthesis and protects cells from oxidative stress: Relevance to Parkinson disease. J. Biol. Chem..

[338] Sacino A.N., Brooks M., Thomas M.A., McKinney A.B., Lee S., Regenhardt R.W., McGarvey N.H., Ayers J.I., Notterpek L., Borchelt D.R. (2014). Intramuscular injection of α-synuclein induces CNS α-synuclein pathology and a rapid-onset motor phenotype in transgenic mice. Proc. Natl. Acad. Sci. USA.

[339] Sacino A.N., Brooks M., McKinney A.B., Thomas M.A., Shaw G., Golde T.E., Giasson B.I. (2014). Brain injection of α-synuclein induces multiple proteinopathies, gliosis, and a neuronal injury marker. J. Neurosci. Off. J. Soc. Neurosci..

[340] Canal M., Martín-Flores N., Pérez-Sisqués L., Romaní-Aumedes J., Altas B., Man H.-Y., Kawabe H., Alberch J., Malagelada C. (2016). Loss of NEDD4 contributes to RTP801 elevation and neuron toxicity: Implications for Parkinson’s disease. Oncotarget.

[341] Pérez-Sisqués L., Sancho-Balsells A., Solana-Balaguer J., Campoy-Campos G., Vives-Isern M., Soler-Palazón F., Anglada-Huguet M., López-Toledano M.-Á., Mandelkow E.-M., Alberch J. (2021). RTP801/REDD1 contributes to neuroinflammation severity and memory impairments in Alzheimer’s disease. Cell Death Dis..

[342] Falkenburger B.H., Saridaki T., Dinter E. (2016). Cellular models for Parkinson’s disease. J. Neurochem..

[343] Xicoy H., Brouwers J.F., Kalnytska O., Wieringa B., Martens G.J.M. (2020). Lipid Analysis of the 6-Hydroxydopamine-Treated SH-SY5Y Cell Model for Parkinson’s Disease. Mol. Neurobiol..

[344] Amo T., Oji Y., Saiki S., Hattori N. (2019). Metabolomic analysis revealed mitochondrial dysfunction and aberrant choline metabolism in MPP+-exposed SH-SY5Y cells. Biochem. Biophys. Res. Commun..

[345] Amo T., Oji Y., Saiki S., Hattori N. (2021). Metabolomic analysis data of MPP+-exposed SH-SY5Y cells using CE-TOFMS. Data Brief.

[346] Rousseaux M.W.C., Marcogliese P.C., Qu D., Hewitt S.J., Seang S., Kim R.H., Slack R.S., Schlossmacher M.G., Lagace D.C., Mak T.W. (2012). Progressive dopaminergic cell loss with unilateral-to-bilateral progression in a genetic model of Parkinson disease. Proc. Natl. Acad. Sci. USA.

[347] Van Rompuy A.-S., Lobbestael E., Van der Perren A., Van den Haute C., Baekelandt V. (2014). Long-term overexpression of human wild-type and T240R mutant Parkin in rat substantia nigra induces progressive dopaminergic neurodegeneration. J. Neuropathol. Exp. Neurol..

[348] Gao H.-M., Zhang F., Zhou H., Kam W., Wilson B., Hong J.-S. (2011). Neuroinflammation and α-synuclein dysfunction potentiate each other, driving chronic progression of neurodegeneration in a mouse model of Parkinson’s disease. Environ. Health Perspect..

[349] Vaz R.L., Sousa S., Chapela D., van der Linde H.C., Willemsen R., Correia A.D., Outeiro T.F., Afonso N.D. (2020). Identification of antiparkinsonian drugs in the 6-hydroxydopamine zebrafish model. Pharmacol. Biochem. Behav..

[350] Carvalho M.M., Campos F.L., Coimbra B., Pêgo J.M., Rodrigues C., Lima R., Rodrigues A.J., Sousa N., Salgado A.J. (2013). Behavioral characterization of the 6-hydroxidopamine model of Parkinson’s disease and pharmacological rescuing of non-motor deficits. Mol. Neurodegener..

[351] Masilamoni G.J., Smith Y. (2018). Chronic MPTP administration regimen in monkeys: A model of dopaminergic and non-dopaminergic cell loss in Parkinson’s disease. J. Neural Transm..

[352] Bonnet A.-M. (2000). Involvement of Non-Dopaminergic Pathways in Parkinson’s Disease. CNS Drugs.

[353] Levy R., Herrero M.T., Ruberg M., Villares J., Faucheux B., Guridi J., Guillen J., Luquin M.R., Javoy-Agid F., Obeso J.A. (1995). Effects of nigrostriatal denervation and L-dopa therapy on the GABAergic neurons in the striatum in MPTP-treated monkeys and Parkinson’s disease: An in situ hybridization study of GAD67 mRNA. Eur. J. Neurosci..

[354] Ullrich C., Humpel C. (2009). Rotenone induces cell death of cholinergic neurons in an organotypic co-culture brain slice model. Neurochem. Res..

[355] Cui K., Yang F., Tufan T., Raza M.U., Zhan Y., Fan Y., Zeng F., Brown R.W., Price J.B., Jones T.C. (2021). Restoration of Noradrenergic Function in Parkinson’s Disease Model Mice. ASN Neuro.

[356] Zhao F., Li C., Zhuang Y., Yan Y., Gao Y., Behnisch T. (2024). Apoptosis signal-regulating kinase 1 (Ask1) deficiency alleviates MPP+-induced impairment of evoked dopamine release in the mouse hippocampus. Front. Cell. Neurosci..

[357] Kroener S., Chandler L.J., Phillips P.E.M., Seamans J.K. (2009). Dopamine modulates persistent synaptic activity and enhances the signal-to-noise ratio in the prefrontal cortex. PLoS ONE.

[358] Wang Y., Qu L., Wang X.-L., Gao L., Li Z.-Z., Gao G.-D., Yang Q. (2015). Firing pattern modulation through SK channel current increase underlies neuronal survival in an organotypic slice model of Parkinson’s disease. Mol. Neurobiol..

[359] Guo S., Lei Q., Yang Q., Chen R. (2024). IGFBP5 Promotes Neuronal Apoptosis in a 6-OHDA-Toxicant Model of Parkinson’s Disease by Inhibiting the Sonic Hedgehog Signaling Pathway. Med. Princ. Pract..

[360] Gaceb A., Barbariga M., Paul G. (2020). An In Vitro Partial Lesion Model of Differentiated Human Mesencephalic Neurons: Effect of Pericyte Secretome on Phenotypic Markers. J. Mol. Neurosci. MN.

[361] Huang C., Ma J., Li B.-X., Sun Y. (2019). Wnt1 silencing enhances neurotoxicity induced by paraquat and maneb in SH-SY5Y cells. Exp. Ther. Med..

[362] Zhang X.-M., Yin M., Zhang M.-H. (2014). Cell-based assays for Parkinson’s disease using differentiated human LUHMES cells. Acta Pharmacol. Sin..

[363] Ji L.-L., Huang T.-T., Mao L.-L., Xu Y.-F., Chen W.-Y., Wang W.-W., Wang L.-H. (2023). The gut microbiota metabolite butyrate mitigates MPTP/MPP+-induced Parkinson’s disease by inhibiting the JAK2/STAT3 signaling pathway. Kaohsiung J. Med. Sci..

[364] Chen Q., Huang X., Li R. (2018). lncRNA MALAT1/miR-205-5p axis regulates MPP+-induced cell apoptosis in MN9D cells by directly targeting LRRK2. Am. J. Transl. Res..

[365] Stępkowski T.M., Wasyk I., Grzelak A., Kruszewski M. (2015). 6-OHDA-Induced Changes in Parkinson’s Disease-Related Gene Expression are not Affected by the Overexpression of PGAM5 in In Vitro Differentiated Embryonic Mesencephalic Cells. Cell. Mol. Neurobiol..

[366] Alegre-Cortés E., Muriel-González A., Canales-Cortés S., Uribe-Carretero E., Martínez-Chacón G., Aiastui A., López de Munain A., Niso-Santano M., Gonzalez-Polo R.A., Fuentes J.M. (2020). Toxicity of Necrostatin-1 in Parkinson’s Disease Models. Antioxidants.

[367] Ito K., Eguchi Y., Imagawa Y., Akai S., Mochizuki H., Tsujimoto Y. (2017). MPP+ induces necrostatin-1- and ferrostatin-1-sensitive necrotic death of neuronal SH-SY5Y cells. Cell Death Discov..

[368] Xu Z., Patterson T.A., Wren J.D., Han T., Shi L., Duhart H., Ali S.F., Slikker W. (2005). A microarray study of MPP+-treated PC12 Cells: Mechanisms of toxicity (MOT) analysis using bioinformatics tools. BMC Bioinform..

[369] Prediger R.D.S., Rial D., Medeiros R., Figueiredo C.P., Doty R.L., Takahashi R.N. (2009). Risk is in the Air. Ann. N. Y. Acad. Sci..

[370] Rodrigues L.S., Targa A.D.S., Noseda A.C.D., Aurich M.F., Da Cunha C., Lima M.M.S. (2014). Olfactory impairment in the rotenone model of Parkinson’s disease is associated with bulbar dopaminergic D2 activity after REM sleep deprivation. Front. Cell. Neurosci..

[371] Pan-Montojo F., Anichtchik O., Dening Y., Knels L., Pursche S., Jung R., Jackson S., Gille G., Spillantini M.G., Reichmann H. (2010). Progression of Parkinson’s disease pathology is reproduced by intragastric administration of rotenone in mice. PLoS ONE.

[372] Visanji N.P., Brooks P.L., Hazrati L.-N., Lang A.E. (2013). The prion hypothesis in Parkinson’s disease: Braak to the future. Acta Neuropathol. Commun..

[373] Doty R.L. (2012). Olfaction in Parkinson’s disease and related disorders. Neurobiol. Dis..

[374] Arshamian A., Iravani B., Lundström J.N. (2022). Is congenital anosmia protective for Parkinson’s disease triggered by pathogenic entrance through the nose?. Npj Park. Dis..

[375] Bagnoli E., Trotier A., McMahon J., Quinlan L.R., Biggs M., Pandit A., FitzGerald U. (2023). Prodromal Parkinson’s disease and the catecholaldehyde hypothesis: Insight from olfactory bulb organotypic cultures. FASEB J..

[376] Dawson T.M., Ko H.S., Dawson V.L. (2010). Genetic Animal Models of Parkinson’s Disease. Neuron.

[377] Sallinen V., Kolehmainen J., Priyadarshini M., Toleikyte G., Chen Y.-C., Panula P. (2010). Dopaminergic cell damage and vulnerability to MPTP in Pink1 knockdown zebrafish. Neurobiol. Dis..

[378] Lev N., Barhum Y., Ben-Zur T., Melamed E., Steiner I., Offen D. (2013). Knocking Out DJ-1 Attenuates Astrocytes Neuroprotection Against 6-Hydroxydopamine Toxicity. J. Mol. Neurosci..

[379] Xia N., Cabin D.E., Fang F., Reijo Pera R.A. (2022). Parkinson’s Disease: Overview of Transcription Factor Regulation, Genetics, and Cellular and Animal Models. Front. Neurosci..

[380] Mata I.F., Lockhart P.J., Farrer M.J. (2004). Parkin genetics: One model for Parkinson’s disease. Hum. Mol. Genet..

[381] Frank-Cannon T.C., Tran T., Ruhn K.A., Martinez T.N., Hong J., Marvin M., Hartley M., Treviño I., O’Brien D.E., Casey B. (2008). Parkin Deficiency Increases Vulnerability to Inflammation-Related Nigral Degeneration. J. Neurosci..

[382] Wang A., Zhong G., Ying M., Fang Z., Chen Y., Wang H., Wang C., Liu C., Guo Y. (2024). Inhibition of NLRP3 inflammasome ameliorates LPS-induced neuroinflammatory injury in mice via PINK1/Parkin pathway. Neuropharmacology.

[383] Milanese C., Sager J.J., Bai Q., Farrell T.C., Cannon J.R., Greenamyre J.T., Burton E.A. (2012). Hypokinesia and Reduced Dopamine Levels in Zebrafish Lacking β- and γ1-Synucleins. J. Biol. Chem..

[384] Robea M.-A., Balmus I.-M., Ciobica A., Strungaru S., Plavan G., Gorgan L.D., Savuca A., Nicoara M. (2020). Parkinson’s Disease-Induced Zebrafish Models: Focussing on Oxidative Stress Implications and Sleep Processes. Oxid. Med. Cell. Longev..

[385] O’Donnell K.C., Lulla A., Stahl M.C., Wheat N.D., Bronstein J.M., Sagasti A. (2014). Axon degeneration and PGC-1α-mediated protection in a zebrafish model of α-synuclein toxicity. Dis. Model. Mech..

[386] Chia S.J., Tan E.-K., Chao Y.-X. (2020). Historical Perspective: Models of Parkinson’s Disease. Int. J. Mol. Sci..

[387] Volpicelli-Daley L.A., Abdelmotilib H., Liu Z., Stoyka L., Daher J.P.L., Milnerwood A.J., Unni V.K., Hirst W.D., Yue Z., Zhao H.T. (2016). G2019S-LRRK2 Expression Augments α-Synuclein Sequestration into Inclusions in Neurons. J. Neurosci..

[388] Bose A., Petsko G.A., Studer L. (2022). Induced pluripotent stem cells: A tool for modeling Parkinson’s disease. Trends Neurosci..

[389] Hunter R.L., Dragicevic N., Seifert K., Choi D.Y., Liu M., Kim H.-C., Cass W.A., Sullivan P.G., Bing G. (2007). Inflammation induces mitochondrial dysfunction and dopaminergic neurodegeneration in the nigrostriatal system. J. Neurochem..

[390] Hunter R., Ojha U., Bhurtel S., Bing G., Choi D.-Y. (2017). Lipopolysaccharide-induced functional and structural injury of the mitochondria in the nigrostriatal pathway. Neurosci. Res..

[391] Hunter R.L., Cheng B., Choi D.-Y., Liu M., Liu S., Cass W.A., Bing G. (2009). Intrastriatal lipopolysaccharide injection induces parkinsonism in C57/B6 mice. J. Neurosci. Res..

[392] Ling Z., Zhu Y., wai Tong C., Snyder J.A., Lipton J.W., Carvey P.M. (2006). Progressive dopamine neuron loss following supra-nigral lipopolysaccharide (LPS) infusion into rats exposed to LPS prenatally. Exp. Neurol..

[393] Capuano A.W., Wilson R.S., Honer W.G., Petyuk V.A., Leurgans S.E., Yu L., Gatchel J.R., Arnold S., Bennett D.A., Arvanitakis Z. (2019). Brain IGFBP-5 Modifies the Relation of Depressive Symptoms to Decline in Working Memory in Older Persons. J. Affect. Disord..

[394] Lewitt M.S., Boyd G.W. (2024). Role of the Insulin-like Growth Factor System in Neurodegenerative Disease. Int. J. Mol. Sci..

[395] Yu H., Sun T., He X., Wang Z., Zhao K., An J., Wen L., Li J.-Y., Li W., Feng J. (2022). Association between Parkinson’s Disease and Diabetes Mellitus: From Epidemiology, Pathophysiology and Prevention to Treatment. Aging Dis..

[396] Li X., Li C., Zhang W., Wang Y., Qian P., Huang H. (2023). Inflammation and aging: Signaling pathways and intervention therapies. Signal Transduct. Target. Ther..

[397] Calabrese V., Santoro A., Monti D., Crupi R., Di Paola R., Latteri S., Cuzzocrea S., Zappia M., Giordano J., Calabrese E.J. (2018). Aging and Parkinson’s Disease: Inflammaging, neuroinflammation and biological remodeling as key factors in pathogenesis. Free Radic. Biol. Med..

[398] Russo T., Riessland M. (2022). Age-Related Midbrain Inflammation and Senescence in Parkinson’s Disease. Front. Aging Neurosci..

[399] Yang L., Zhou R., Tong Y., Chen P., Shen Y., Miao S., Liu X. (2020). Neuroprotection by dihydrotestosterone in LPS-induced neuroinflammation. Neurobiol. Dis..

